# Biocontrol of Bacterial Leaf Blight of Rice and Profiling of Secondary Metabolites Produced by Rhizospheric *Pseudomonas aeruginosa* BRp3

**DOI:** 10.3389/fmicb.2017.01895

**Published:** 2017-09-26

**Authors:** Sumera Yasmin, Fauzia Y. Hafeez, Muhammad S. Mirza, Maria Rasul, Hafiz M. I. Arshad, Muhammad Zubair, Mazhar Iqbal

**Affiliations:** ^1^Soil and Environmental Biotechnology Division, National Institute for Biotechnology and Genetic Engineering, Faisalabad, Pakistan; ^2^Department of Biosciences, COMSATS Institute of Information Technology, Islamabad, Pakistan; ^3^Plant Protection Division, Nuclear Institute of Agriculture and Biology, Faisalabad, Pakistan

**Keywords:** *Xanthomonas oryzae*, super basmati, mass spectroscopy, HAQ, CLSM, BLB

## Abstract

*Xanthomonas oryzae* pv. *oryzae* (Xoo) is widely prevalent and causes Bacterial Leaf Blight (BLB) in Basmati rice grown in different areas of Pakistan. There is a need to use environmentally safe approaches to overcome the loss of grain yield in rice due to this disease. The present study aimed to develop inocula, based on native antagonistic bacteria for biocontrol of BLB and to increase the yield of Super Basmati rice variety. Out of 512 bacteria isolated from the rice rhizosphere and screened for plant growth promoting determinants, the isolate BRp3 was found to be the best as it solubilized 97 μg/ mL phosphorus, produced 30 μg/mL phytohormone indole acetic acid and 15 mg/ L siderophores *in vitro*. The isolate BRp3 was found to be a *Pseudomonas aeruginosa* based on 16S rRNA gene sequencing (accession no. HQ840693). This bacterium showed antagonism *in vitro* against different phytopathogens including Xoo and *Fusarium* spp. Strain BRp3 showed consistent pathogen suppression of different strains of BLB pathogen in rice. Mass spectrometric analysis detected the production of siderophores (1-hydroxy-phenazine, pyocyanin, and pyochellin), rhamnolipids and a series of already characterized 4-hydroxy-2-alkylquinolines (HAQs) as well as novel 2,3,4-trihydroxy-2-alkylquinolines and 1,2,3,4-tetrahydroxy-2-alkylquinolines in crude extract of BRp3. These secondary metabolites might be responsible for the profound antibacterial activity of BRp3 against Xoo pathogen. Another contributing factor toward the suppression of the pathogen was the induction of defense related enzymes in the rice plant by the inoculated strain BRp3. When used as an inoculant in a field trial, this strain enhanced the grain and straw yields by 51 and 55%, respectively, over non-inoculated control. Confocal Laser Scanning Microscopy (CLSM) used in combination with immunofluorescence marker confirmed *P. aeruginosa* BRp3 in the rice rhizosphere under sterilized as well as field conditions. The results provide evidence that novel secondary metabolites produced by BRp3 may contribute to its activity as a biological control agent against Xoo and its potential to promote the growth and yield of Super Basmati rice.

## Introduction

Rice is an important staple food crop. The global production of rice paddy was 746.9 million tons and 496 million tons milled rice (http://www.fao.org/economic/est/publications/rice-publications/rice-market-monitor-rmm/en/). The crop is widespread all over the world due to its wider adaptability under different environmental conditions. For this reason, Food and Agriculture Organization (FAO) regarded it as a strategic crop for food security in the world (Montano et al., 2014). Rice is used as a staple food in different areas of world especially in Asia (http://www.britannica.com/plant/rice). In Pakistan, beside wheat, rice is the 2nd major cereal crop, which is widely cultivated, consumed, and exported. Among various cultivated varieties, Super Basmati rice variety is the most sought variety, as it is very popular among consumers, farmers, traders, and exporters due to its high quality grain and aroma (http://reap.com.pk/news/news_detail.asp?newsid=3905). However, susceptibility of this variety to different diseases is a major problem. Among these, *Xanthomonas oryzae* pv. *oryzae*, the causal agent of bacterial leaf blight (BLB) is considered to cause severe yield losses (Arshad et al., 2015). This disease is widely prevalent among various rice varieties worldwide (Singh et al., 2015). Historically, BLB was initially reported in Japan during 1884–1885 and then reported in other rice growing countries (Gnanamanickam, 2009). Rice crop was severely affected by this disease in areas of tropica and Asia with heavy rainfall in monsoon. Initially, its occurrence in Pakistan was reported by Mew and Majid (1977) and Arshad et al. (2015). Later studies indicated an alarming increase in BLB incidence in Basmati rice growing areas of the country (Shah et al., 2009).

BLB occurs at different growth stages of rice and is manifested by either leaf blight or “Kresek” (acute wilting of young plants) symptoms. Xoo invades the plant through wounds or water pores. Lesions with wavy margins start from the tip of the leaf as the water pores are located at the margins of upper parts of the leaf. These water soaked lesions enlarge in size, turn yellow and ultimately lead to the death of plant (Nino-Liu et al., 2006).

In the past, various disease management strategies have been employed to reduce the yield losses and to avoid disease epidemics but use of chemicals has not been successful due to variation in sensitivity of pathogenic races toward applied chemicals. Development of mutation in pathogenic races is a major hindrance in developing a durable control (George et al., 1997). Moreover, due to toxic residues, usage of antibiotics and chemicals against rice BLB, has limitations (MacManus et al., 2002). Although, the use of host resistance genes seems to be practicable, single gene (Xa4) based breeding for BLB management has been shown to be ineffective due to evolution of sub-populations that overcome these resistance genes (Shanti et al., 2010). As a result, biological control seems to be a cost effective and environmentally friendly way to manage this serious threat (Gnanamanickam, 2009).

Most of the rhizospheric antagonistic bacteria such as *Pseudomonas* spp. can indirectly increase plant resistance by improving the plant growth. Responses of the host plant are due to root colonization of a plant by antagonistic rhizobacteria that play an important role in disease suppression. *Lysobacter antibioticus* have been documented as biocontrol agents against Xoo due to their rapid growth, easy application and effective leaf colonization (Ji et al., 2008). Plant growth promoting *Bacillus* spp. were found to suppress BLB in rice under greenhouse conditions (Chithrashree et al., 2011). Li et al. (2011) reported *Streptomyces globisporus* for the suppression of rice blast caused by *Magnaporthe oryzae*. The incidence of sheath blight was reduced by some biofilm forming and surfactant producing strains of *Bacillus subtilis* (Mousivand et al., 2012). *Streptomyces philanthi* and a commercial formulation of *B. subtilis* were found to be biologically active against rice sheath blight when integrated with chemical fungicides (Boukaew et al., 2013). Hydrogen cyanide (HCN) producing *Pseudomonas chlororaphis* significantly inhibited the growth of *M. oryzae*, showing its biocontrol properties against the causal agent of rice blast (Spence et al., 2014).

These antagonistic bacteria can directly suppress plant pathogens by producing antibiotics, enzymes like chitinases, glucanases, proteases, and siderophores or indirect mechanisms in which the antagonistic bacteria compete with the pathogen for a niche or nutrient sites (Bardin et al., 2015). These bacteria have been reported to reduce the disease incidence significantly under controlled as well as under natural field conditions. *Bacillus* and *Pseudomonas* spp. control the diseases caused by rice pathogens i.e., *X. oryzae* pv. *oryzae, Rhizoctonia solani*, and *M. oryzae* up to 90% depending on the bacteria used, pathogen and the rice variety (Montano et al., 2014). Induced systemic resistance (ISR) is an environmentally attractive option for disease control whereby plant defenses are enhanced as a result of their interaction with certain rhizobacteria. ISR contributes positively toward the biological control of plant pathogens and the defense related enzymes induced by the inoculated bacteria protect the host plants (Chithrashree et al., 2011). Systemic resistance was induced by *Serratia* sp. causing resistance against necrotrophic leaf pathogens in rice (Vleesschauwer et al., 2009).

However, most of the previous studies investigated either plant growth promoting activities or biocontrol activities of the bacterial isolates, exclusively. Previously, Kumar et al. (2005) and Fang et al. (2013) reported broad spectrum antifungal and biofertilizer activity of *Pseudomonas aeroginosa* and *Pseudomonas aurantiaca*. Similarly, different endophytic strains of *B. subtilis* have been reported to have plant growth promoting activity on cacao and biocontrol activity against phytopathogens like *Moniliophthora perniciosa* and *Colletotrichum* spp. but the antifungal metabolites were not investigated (Kumar et al., 2012; Nain et al., 2012; Falcao et al., 2014). The use of *Pseudomonas* and *Bacillus* strains have been reported for the biocontrol of rice pathogens such as Xoo, *M. oryzae*, and *R. solani* (Ji et al., 2008; Helene et al., 2011; Spence et al., 2014). However, again the major emphasis of these previous studies was on the biocontrol activity. Rhizospheric antagonistic *Pseudomonas aeruginosa* have been documented as beneficial biocontrol agents against Xoo (Yasmin et al., 2016) but the role of diverse secondary metabolites produced by different strains of *P. aeruginosa* in the suppression of BLB pathogen has not been reported earlier.

Members of the *Pseudomonas* spp., including pathogenic as well as non-pathogenic strains, are capable of producing various extracellular secondary metabolites. These metabolites exhibit diverse properties i.e., function as virulence factors, siderophores (having high-affinity of iron ions), biosurfactants, and antimicrobial agents as well as in cell-to-cell signaling etc. These metabolites enable *Pseudomonas* spp. to adapt in different environments, colonize different hosts and compete with other species. *Pseudomonas* metabolites constitute chemical entities such as N-acylhomoserine lactone (Cataldi et al., 2009), phenazine (Price-Whelan et al., 2006; Kumar et al., 2011; Jain and Pandey, 2016), pyochellin (Youard et al., 2007; Jayaseelan et al., 2014), phloroglucinol (Kidarsa et al., 2011), lahorenoic acid, diketopiperazines (Mehnaz et al., 2013), 4-hydroxy-2-alkylquinolines (Lepine et al., 2004), rhamnolipids (Soberon-Chavez et al., 2005; Grosso-Becerra et al., 2016), and cyclic lipopeptides (Mehnaz et al., 2013). Knowing the chemistries of these metabolites can help with characterization of their biological and physiological activities. However, most of the existing studies have been confined to the analysis of individual metabolite class analysis and its biological properties (Kumar et al., 2005). There are few studies where more than one class of metabolites have been analyzed simultaneously. Even during the study of one physiological aspect like bacterial quorum-sensing, analysis of multiclass metabolites [N-acyl-L-homoserine lactone and 2-alkyl 4-(1H)-quinolone] can enhance the scope of study (Ortori et al., 2011). In this study, detailed chemical characterization of the BRp3 supernatant has led to identification of a variety of previously known as well as novel metabolites of several chemical classes.

Despite the economic importance of BLB, complete resistance to this disease has not been reported. Furthermore, no local resistant varieties are commercially available in Pakistan. Therefore, to select bacteria with multiple beneficial applications for the improvement of rice crop, 512 rhizobacteria were isolated from different field sites of Punjab (Pakistan). After screening their plant growth promoting as well as biocontrol activities, the bacterium termed as BRp3, demonstrated the best results in rice yield improvement and biocontrol activity against the prevalent rice pathogen Xoo. This bacterium was subjected to detailed characterization of the secondary metabolites with ultimate aim to develop inocula of functionally well-characterized native antagonistic bacteria, having a “dual-purpose inoculum” with strong plant growth promoting and biocontrol aspects, for yield enhancement of “Super Basmati” rice variety.

Mass spectrometric analysis revealed the production of siderophores, rhamnolipids, a series of previously known and novel HAQs in the crude extract of BRp3. These results high-lighted the facts that *P. aeroginosa* BRp3, owing to its capability to produce a number of secondary metabolites in HAQ, rhamnolipids, and siderophores (phenazines and pyochellin) series, exhibited intense antimicrobial activity against Xoo pathogens. Hence, this bacterium could be used as an effective biocontrol agent against the rice pathogen Xoo.

## Materials and methods

### Bacterial pathogens used

Six *X. oryzae* pv. *oryzae* (Xoo) strains i.e., Xoo1, Xoo2, Xoo4, Xoo5, Xoo6, and Xoo7 were obtained from NIBGE Biotech Resource Centre (NBRC), Faisalabad (Yasmin et al., 2016). The pathogenicity of Xoo strains was tested by clip inoculation (Kauffman et al., 1973) on three susceptible rice varieties i.e., Super Basmati, Basmati 385, and Basmati 2000. Clip inoculation of Xoo1, Xoo2, Xoo4, Xoo5, and Xoo6 to rice showed typical symptoms of BLB on the inoculated leaves i.e., white to gray lesions starting from leaf tip to downward along leaf veins and margins. Bacterial Xoo pathogens were stored in PSA agar (Ou, 1985) slants at 4°C and in 20% glycerol at −80°C for further use.

### Isolation of rhizobacteria with biocontrol activity

Root samples of healthy rice plants in the vicinity of BLB infected plants were collected from fields of different rice growing sites. These root samples with adhering soil were used to isolate bacteria from rhizosphere, rhizoplane, and endosphere on Nutrient Agar (Norris and Ribbons, 1970), King's B (King et al., 1954), and Gould's S1 medium (Gould et al., 1985) using serial dilution method as mentioned by Somasegaran and Hoben (1994). Purified bacterial strains were stored in LB agar slants at 4°C and in 20% glycerol at −80°C.

The rhizobacteria were screened *in vitro* for inhibition of Xoo using plate diffusion method (Hewitt and Vincent, 1989). A fresh culture (100 μL) of Xoo grown in PSA broth was spread onto LB plates. The liquid culture of bacterial strains grown in LB broth to be tested for antagonistic activity was spotted on LB plates already spread with Xoo strain. These plates were kept in incubator at 30 ± 2°C for 48 h and the antibiosis was observed by measuring the zone of inhibition of pathogen's growth (Velusamy et al., 2006).

Antagonistic activity of *P. aeruginosa* BRp3 was tested against phytopathogenic fungi like *Fusarium oxysporum, Fusarium monoliforme*, and *Fusarium solani* by dual culture assay on Potato Dextrose agar (PDA; Ji et al., 2008) and the percent inhibition was calculated (Yasmin et al., 2014).

### Primary selection of antagonistic bacteria for growth promotion

Primary selection of antagonist was carried out based on its antagonism as well as its effect on rice (Super Basmati) seedlings in a growth room experiment. The seeds were disinfected with sodium hypochlorite (1%) for 5 min and then washed thrice with sterilized water. Twenty seeds soaked in over-night grown bacterial broth culture of antagonistic strain (log phase containing 10^9^ CFU mL^−1^), were grown aseptically on sterile wet filter paper each kept in each sterile Petri plate (14 cm diameter). Twenty-four bacteria were selected and there were three replicates for each treatment. Un-inoculated seedlings were used as control. The plates were kept in a growth room and maintained at a day/night temperature of 30 ± 2°C/25 ± 2°C and 16 h day length with 20,000 Lux light intensity. The plates were watered with sterilized distilled water in laminar flow. The number of germinated seedlings, radical length and hypocotyl length were measured after 10 days.

### Identification by 16S rRNA gene sequencing and phylogenetic analysis

CTAB method (Ausubel et al., 1992) was used to extract total genomic DNA of strain BRp3. Universal primers P1 and P6 were used to amplify 16S rRNA gene (Tan et al., 1997). QIAquick Gel Extraction Kit (QIAGEN Sciences, Maryland 20874, USA) was used to clean the amplified PCR product about 1.5 Kb of 16S rRNA gene. Amplified PCR products were commercially sequenced by Macrogen, Inc. (Seoul, South Korea). Available sequences of bacterial lineage in NCBI were used to align and compare the sequence data using BLAST. Accession numbers were allocated after submitting the sequences to NCBI GenBank database (Yasmin et al., 2016).

For calculating phylogenetic tree of strain BRp3, closely related sequences were downloaded and aligned using CLUSTAL X and MacClade 4.05 (Thompson et al., 1997; Maddison and Maddison, 1999). Maximum Parsimony (MP), maximum likelihood (ML) and neighbor joining (NJ) methods were used for sequences analysis as mentioned by Mirza et al. (2009).

### Detection of growth promoting and biocontrol determinants

Bacterial nitrogen fixation was estimated by using acetylene reduction assay (ARA; Hardy et al., 1968). Phosphate solubilization by the bacterium was measured on Pikovskaya agar medium added with insoluble tricalcium phosphate and quantified by Phospho-molybdate blue color method (Murphy and Riley, 1962). Quantification of produced Indole Acetic Acid (IAA) was carried out following the method of Tien et al. (1979). 1-Aminocyclopropane-1-carboxylic acid (ACC) deaminase activity was assessed in vials added with 3 μL of ACC (0.5 M) as a sole N source in 5 mL DF salt minimal medium (Penrose and Glick, 2003).

Production of siderophores was observed on universal chrome azurol “S” (CAS) agar medium (Schwyn and Neilands, 1987) and quantified as described by Rachid and Bensoltane (2005). Production of HCN was detected following the method as described by Lork (1948). Protease and chitinase activities were detected on skim milk agar (Denizci et al., 2004) and chitin agar (Brien and Colwall, 1987) media. Glucanolytic activity was detected on minimal medium supplemented with glucan source i.e., Lamimarin containing 0.5% yeast extract (Qing et al., 2002). Starch hydrolyzing ability was detected on nutrient agar added with 2% starch (Marten et al., 2000). There were three biological replicates for all the tested growth promoting and biocontrol determinants.

Antibiotic resistance of the bacterial strain BRp3 was determined on antibiotic sensitivity sulfonamide (ASS) agar medium by disc diffusion method (Valverde et al., 2005) using commercial antibiotic susceptibility discs (Bioanalyse, Turkey; Imran et al., 2015).

### Mass spectrometric analysis of culture supernatant

For the detection and identification of secondary metabolites, *P. aeruginosa* BRp3 was inoculated to LB medium (500 mL) and incubated with shaking at 30 ± 2°C for 24 and 48 h. To remove the bacterial cell pellet, the bacterial culture was centrifuged at 4°C for 15 min at 10,000 rpm. pH of the supernatant was reduced to 3.0 using 3M HCl solution before its extraction with 500 mL ethyl acetate. This extraction step was repeated using 300 mL ethyl acetate. The combined organic layers were obtained and evaporated under reduced pressure by rotary evaporation. Residues were dissolved in 5 mL LCMS grade methanol and subjected to LCMS/MS analysis using mass spectrometer (LTQ XL Linear Ion Trap Mass Spectrophotometer, Thermo Scientific, USA), equipped with an ESI source. The samples were filter sterilized and were injected through direct syringe pump with a flow rate of 8 μL min^−1^. Samples were scanned at both positive and negative total ion full scan modes (mass scan range *m*/*z* 50–2,000) with source voltage and capillary voltage of 4.8 kV and 23 V, respectively. Capillary temperature and sheath glass flow (N_2_) were 350°C and 30 arbitrary units, in both scan modes. The selected analytes were fragmented at positive and negative ion modes by employing collision induced dissociation (CID) energy of 35 (percentage of 5 V) or otherwise stated.

The supernatant of the bacterial culture was studied for its antimicrobial activity against Xoo in plate diffusion assay at different time intervals (24–144 h) as described in section Isolation of Rhizobacteria with Biocontrol Activity. The supernatant of the bacterial culture with higher antimicrobial activity (after 24 and 48 h) was further subjected to LCMS analysis for the detection of secondary metabolites.

### *In planta* evaluation for the suppression of BLB

#### Net house experiment

Antagonistic bacteria BRp3 was evaluated *in vivo* for the suppression of BLB against reference strain Xoo2 (selected on the basis of its virulence) under natural light and temperature conditions at NIBGE net house during the rice growing season. The seeds were treated with the antagonistic bacteria (10^9^ CFU mL^−1^) for 2 h and sown in small plastic pots of 12 × 7 cm^2^ diameter filled with 3 kg soil/ pot (texture clay loam, organic matter 0.4%, pH 8.5, EC 4.1 mS, viable cell count 1.8 × 10^6^ cell g^−1^ soil). The experiment was carried out using completely randomized block design (CRD). There were four replicates for each treatment with 15 plants per pot. After 21 days, the plants were given foliar spray treatment with their respective antagonistic bacteria (10^9^ CFU mL^−1^) made in sterilized distilled water. Control plants (healthy control) were sprayed with sterilized water. Antibiotic streptocyclin applied @ 5 mg/pot was used as positive control. Sterilized water was used to clip inoculate the leaves of control plants whereas leaves of inoculated treatments and infected control (IC) were clip inoculated with broth culture of Xoo (10^6^ CFU mL^−1^) on 23rd day of sowing as per the method of Kauffman et al. (1973). Lesion length was measured on 15th day of the inoculation and data for one treatment was obtained from 40 inoculated leaves. The plant dry weight obtained in the inoculated treatments was compared with the non-inoculated healthy control. Suppression of BLB was measured in terms of reduction in the mean bacterial blight lesion length on treated leaves compared to those of non-inoculated control using the following formula:

$$\begin{array}{l} \%\text{Diseased leaf area }(\%\text{DLA})=\\ \;\frac{\text{Total lesion length of the test sample}}{\text{Total leaf length of the test sample}}\times 100 \end{array}$$

Antagonistic bacterium BRp3 was re-evaluated against another reference strain Xoo1 in large earthen pots of 24 cm diameter containing 10 kg soil with same characteristics as mentioned earlier in this section.

#### Induction of defense related enzymes

Rice plants grown under net house condition, applied with foliar spray of BRp3 and clip inoculated with Xoo2 pathogen as described in section Net House Experiment, were also used to study the induction of defense related enzymes (Chithrashree et al., 2011).

Leaves were harvested at 24, 48, 72, and 144 h after application of foliar spray of strain BRp3 on rice plants. There were 15 plants per pot and five leaves were taken randomly from replicates of each treatment. These leaves were mixed thoroughly after cutting them and then 0.1 g sample was used immediately for analysis of different enzymes. Among different defense related enzymes, peroxidase (POD) activity was assayed using guaicol as a substrate at 470 nm wavelength (Hammerschmidt et al., 1982; Liang et al., 2011). Polyphenol oxidase (PPO) was determined at 280 nm using L-tyrosine as the substrate (Worthington, 1988). Decrease in the amount of hydrogen per oxide was determined at 240 nm wavelength to study the catalase (CAT) activity as described previously (Weisany et al., 2012). Phenylalanine ammonia-lyase (PAL) activity was analyzed at 290 nm wavelength by measuring the conversion of L-phenylalanine into trans-cinnamic acid (Benkeblia, 2000).

#### Field experiment

Bacterial strain BRp3 was evaluated under field conditions against BLB using rice variety Super Basmati during crop season June-Oct 2013 in a BLB nursery at Plant Protection Division, NIAB, Faisalabad (a hot spot for BLB incidence). The soil sample was homogenized and used for further analysis (pH 8.2, EC 1.8 mS, 1.3 mg total N g^−1^ soil, 1 μg available P g^−1^ soil, 0.03% organic matter and population density of indigenous bacteria i.e. 1 × 10^6^ cells g^−1^ soil). Seeds were dipped in broth culture of antagonistic bacteria (1 × 10^9^ CFU mL^−1^) for half an hour. Inoculated/un-inoculated seeds were directly sown in seedbeds of 1 m row plots. Each bed was irrigated independently without allowing the water flow from one bed to irrigate another. The beds were separated by a distance of 0.5 m with mud bunds. Each treated row was bordered with a row as a buffer zone. Seeds treated with sterilized LB broth medium were considered as negative controls. Each treatment had three replicates arranged in a Randomized Complete Block Design (RCBD). Plant to plant distance was 22 cm. Application of biocontrol agent and antibiotic i.e., streptocyclin (500 g ha^−1^) was carried out as mentioned earlier for pot experiment. The leaves (60 leaves per treatment) were clip inoculated with Xoo7 at maximum tillering stage. Xoo7 was the prevalent strain isolated in the same season from BLB infected area of Sheikhupura, Pakistan. In addition to disease severity, straw weight, and grain weight were also recorded.

### Field evaluation for yield increase of rice

Another field experiment was conducted during crop season June–Oct 2014 to compare the effects of BRp3 with that of commercial biofertilizer (*BioPower)* on the yield of rice variety Super Basmati with different levels of fertilization. The soil was collected from 10 sites of field (0–200 mm depth) before sowing and after application of fertilizer. The soil sample were homogenized and used for further analysis i.e., pH (8.6), EC (4 mS), total N (2 mg g^−1^ soil), available P (8 μg g^−1^ soil), organic matter (0.1%), and population density of indigenous bacteria (1 × 10^6^ cells g^−1^ soil) by viable cell count. At the time of transplanting, *BioPower* and the bacterial strain BRp3 were inoculated by root dip method (Tariq et al., 2007). Non-inoculated treatments with recommended N and P (140–80 kg NP acre^−1^) and with 80% of the recommended N and P doses were considered as controls of experiment. Each treatment had four replicates in RCBD. Nutrient inputs of N and P were applied as urea and di-ammonium phosphate (DAP), respectively. Complete dose of DAP was applied before sowing and urea was applied in three split doses i.e., first one was added at the time of field preparation, second and third dose after 25 and 50 days of transplantation, respectively. The plot size was 4 × 7 m. Planting distance and row spacing was 22 cm. Plots were weeded by hand after water removal at each fertilizer application. The plants were harvested at maturity. Whole plot grain yield and straw yield was recorded after threshing. Plants were manually harvested and weighed after sun drying. Rice yield was expressed as weight of paddy at 14% water content (Wimberly, 1983).

### Rhizosphere colonization studies

Viable count was used to monitor variations in the population of *P. aeruginosa* BRp3 associated with rice variety Super Basmati. The morphology of the colonies of the antagonistic strain was used as a monitoring tool as it produced green pigments in LB agar plates. The inoculated bacterium was further identified on the basis of its antibiotic resistance pattern, antagonism against Xoo strains, production of IAA and siderophores and P solubilization.

Three root and shoot samples from each replicate field plot were collected 14, 21, 40, and 60 days after treatment. Root and shoot samples were washed with sterile distilled water for few minutes. The shoot parts were crushed in sterilized phosphate buffer (10 mL). The suspension was used for preparing serial dilutions followed by plating on LB agar plates. Population level of strain was measured as log_10_ CFU g^−1^ fresh weight of roots and shoots.

Fluorescent antibodies (FA) against *P. aeruginosa* BRp3 were raised for colonization studies. Immunoglobins were separated and then conjugated with fluorescein isothiocynate (FITC). Un-conjugated dye was separated with a Sephadex G-25 column chromatography following the method of Bilal et al. (1993). The quality and specificity of the FA was determined by staining a number of known cultures of *P. aeruginosa* (Somasegaran and Hoben, 1994).

In a pot experiment, surface sterilized seeds of rice variety Super Basmati were sown in small plastic pots having 50 g air-dried, sieved sterilized sand. The non-inoculated pots were treated independently as control. *P. aeruginosa* BRp3 was seed inoculated (10^9^ CFU mL^−1^). All pots were kept in net house during rice growing season. Plants were harvested 21 days after seed germination. Nonspecific adsorption of stain was suppressed using RhITC conjugate. Specific FA was used to stain the roots (Yasmin et al., 2014). Confocal laser scanning microscope (Olympus FV1000, Japan) facilitated with an Argon-ion laser and FV10-ASW 1.7 imaging software was used to observe the fluorescent bacteria at 488 and 525 nm for absorption and emission of FITC, respectively.

### Statistical analysis

Data obtained from *in vitro* and *in vivo* seed/ plant inoculation experiments was subjected to Analysis of Variance (ANOVA). The treatment means were separated by Duncan's multiple range test (DMRT) for plate/pot experiments at 1% (*P* ≤ 0.01) and field evaluation at 5% (*P* ≤ 0.05) significance level, respectively using “MSTATC” program (Duncan, 1995).

## Results

### Isolation and identification of rhizobacteria with biocontrol activity

Out of 512 bacterial isolates obtained from different host plants, 79 isolates showed antagonistic activity against Xoo (Tables S1, S2). Isolate BRp3 showed maximum growth inhibition of all the tested Xoo strains with zone of inhibition ranged from 10 to 24 mm (Table 1). There were three replicates each time and the experiment was repeated thrice. On PDA medium, BRp3 also showed the inhibition of *F. moniliforme* and *F. solani* up to 43.3 and 75%, respectively (Table S1).

**Table 1:** Growth promoting and biocontrol determinants of rice rhizosphere associated *Pseudomonas aeruginosa*.

**Strain**	**IAA^a^ Sμg mL^−1^**	**P^b^ Sμg mL^−1^**	**Proteases^c^**	**HCN^d^**	**Starch hydrolysis^e^**	**Siderophores production^f^ mg L^−1^**	**Diameter of inhibition of Xoo strains**^g^ **(mm)**						**Effect of bacterial inoculation on rice seedlings in a plate assay (% increase compared to non-inoculated control)**^h^		
							**Xoo1**	**Xoo2**	**Xoo4**	**Xoo5**	**Xoo6**	**Xoo7**	**Germination**	**Radical length**	**Hypocotyl length**
*Pseudomonas aeruginosa* BRp3	30 ± 2	97 ± 4	+++	+++	++	15 ± 1.5	20 ± 1	24 ± 2	10 ± 1	10 ± 1	20 ± 1	12 ± 1	19%	65%	56%

*BRp3^a^. Indole acetic acid detected qualitatively by HPLC*.

b *Phosphate solubilization was quantified was quantified using spectrophotometer*.

c *Protease production was carried out on skim milk agar medium. ++ Represent 5–10 mm halo zone, +++ Represents >10 mm halo zone*.

d *Hydrogen cyanide (HCN) production*.

e *Starch hydrolysis was detected by plate assay, ++ Represents hydrolysis of starch in more than half plate, +++ Represents complete hydrolysis of starch in plate*.

f *Siderophore production was detected on CAS agar and quantified using spectrophotometer*.

g Inhibition of Xanthomonas oryzae pv. oryzae (Xoo) strains was determined by “Diffusion plate assay.”

h *Seed germination assay was carried out in a growth room and the data was recorded after 10 days. There were 20 seeds per plate. Seedlings without inoculation were used as control. All values are an average of three biological replicates, ± standard deviation*.

16S rRNA gene sequencing identified the strain BRp3 as *Pseudomonas* sp. Results of Blast showed 99% homology with 16S rRNA gene of *P. aeruginosa* isolate PM-007 (Accession no. KY908465.1). 16S rRNA gene sequence of selected strain was submitted to GenBank data base and accession number was allocated (HQ840693). 16S rRNA sequence based phylogenetic analysis showed that the bacterium BRp3 had maximum sequence similarity with *P. aeruginosa*. It occupied the same phylogenetic branch as the *P. aeruginosa* group (Figure S1).

### Primary selection of antagonistic bacteria for growth promotion

To study the effect of antagonistic bacteria on seedlings, rice seeds were pretreated with different bacteria. Antagonistic bacteria showed variable effects on germination of rice seeds, radical, and hypocotyl length (Figure S2). Bacterial isolates showing less effect on these growth parameters of the rice variety Super Basmati as compared to the control were excluded from the further study.

### Detection of growth promoting and biocontrol determinants

The bacterial strain BRp3 produced 30 ± 2 μg mL^−1^ IAA when supplemented with tryptophan and solubilized P (97 ± 4 μg mL^−1^) as quantified by spectrophotometer (Table 1). The bacterium did not show the nitrogenase activity but used ACC as carbon source in DF salt minimal medium. BRp3 produced volatile compounds such as HCN as observed by a color change of filter paper from yellow to brown in the plate assay. BRp3 showed the activity of proteases but did not produce chitinases and glucanases. This strain was able to hydrolyze starch and showed siderophores production on CAS blue agar medium with a color change from blue to orange. Quantification of siderophores using spectrophotometer showed the production of 15 ± 1.5 mg L^−1^ siderophores by the strain BRp3 (Table 1; Figure S3).

The tests on antibiotic resistance showed that BRp3 was intrinsically resistant to 12 antibiotics i.e., Amikacin, Aztrreonum, Ampicillin, Carbenicillin, Cephradine, Chloramphenicol, Doxycycline, Erythromycin, Gentamicin, Neomycin, Nalidixic acid, and Streptomycin (Table S3).

### Mass spectrometric analysis of culture supernatant

**Figure 1:**
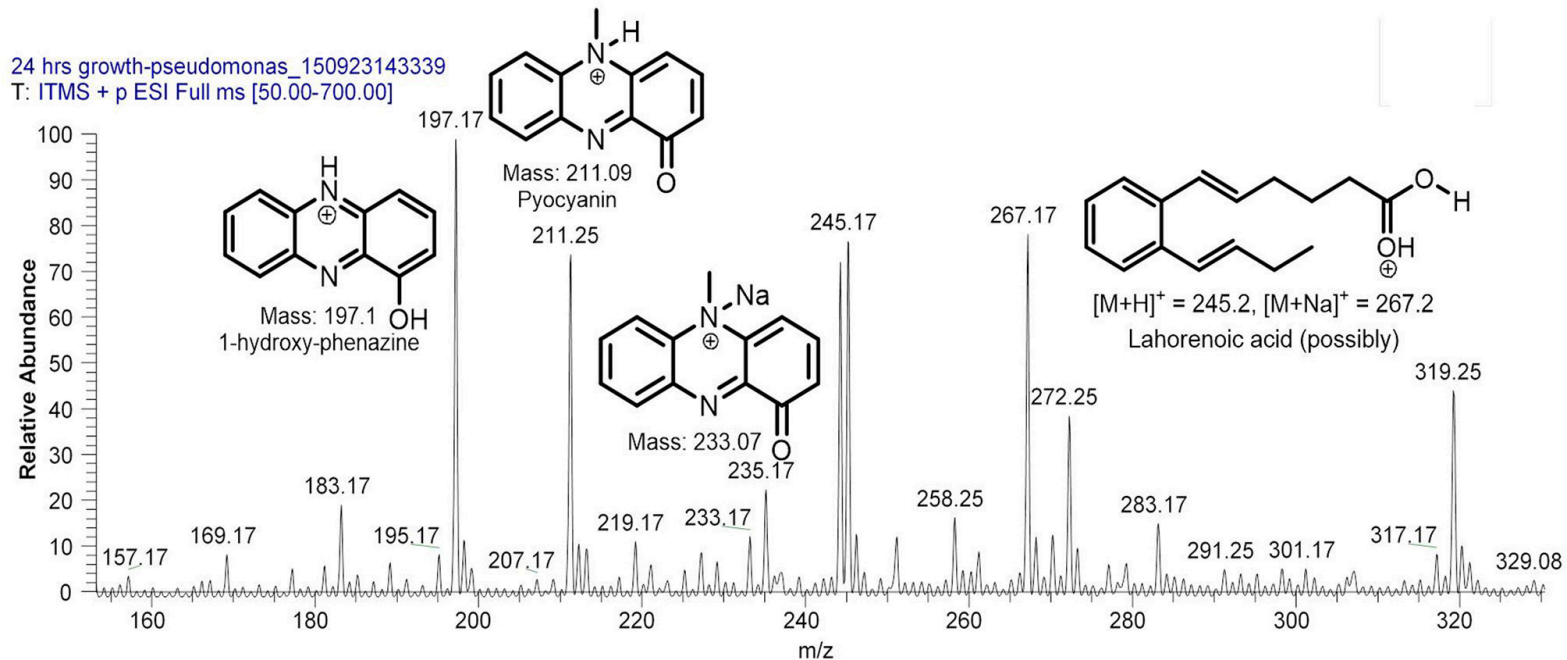
LC/MS chromatogram of *Pseudomonas aeruginosa* BRp3 extract (24 h growth) demonstrating the presence of (predominantly) 1-hydroxy-phenazine, pyocyanin, and possibly lahorenoic acid analyzed at positive ion mode.

**Figure 2:**
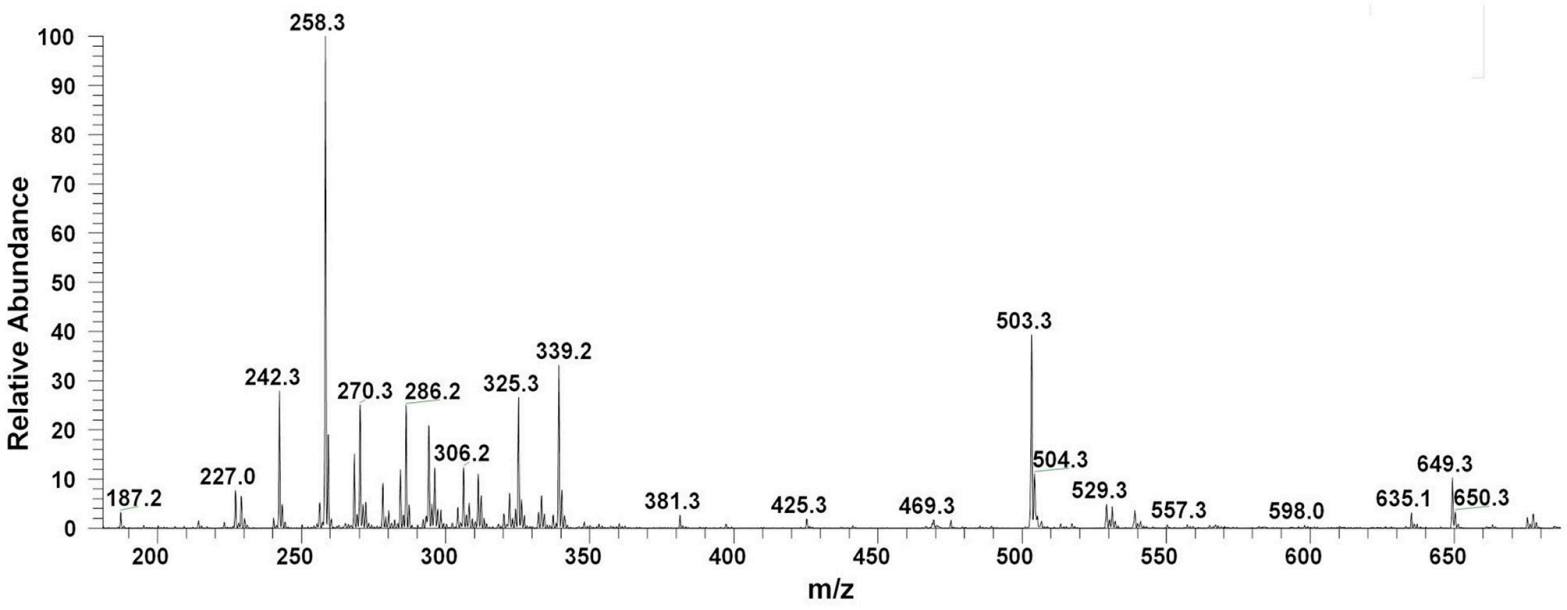
LC/MS chromatograms of *Pseudomonas* extract (48 h growth) indicating the presence of 4-hydroxy-2-alkylquinolines (HAQs) molecular ions species, siderophore (pyochellin, *m*/*z* 325), and rhamnolipids (*m*/*z* 500–650), at negative ion mode analysis.

Filter sterilized cell free extract of *P. aeruginosa* BRp3 was studied by ESI-MS/MS technique. Samples were injected using direct syringe pump and analyzed at both positive as well as negative scan mode. At 24 h growth (after getting the bacteria from culture stock), analysis of crude extract demonstrated the presence of predominant metabolites such as 1-hydroxy-phenazine having *m/z* at 197 [M+H]^+^, 219 [M+Na]^+^; pyocyanin *m/z* at 211 [M+H]^+^, 233 [M+Na]^+^ and possibly lahorenoic acid at *m/z* 245 [M+H]^+^, 267 [M+Na]^+^ (Figure 1). However, this trend altered after the 48 h growth and prominent peaks of 4-hydroxy-2-alkylquinolines (HAQs) exhibiting *m/z* from 214 to 340 [M-H]^−^, siderophore (pyochellin) *m/z* 325 [M-H]^−^, and rhamnolipids *m/z* 503, 529, 539, 635, 649 [M-H]^−^ (Figure 2). The presence of these metabolites was confirmed using tandem mass spectrometry as well as through comparing the values with the literature data (Table 2).

**Table 2:** Metabolites produced by *Pseudomonas aeruginosa* BRp3 detected by ESI-MS/MS.

**S. no**.	**Structures of distinct series of HAQ compounds**	**Side chains**	**Observed peaks (*****m***/***z*****)**			MS/MS (verified)^*^	**MS/MS (reported)**	**References**
			**[M**+**H]**^**−**^		**[M–H]^+^**			
			***m/z***	Abundance^**^				
1	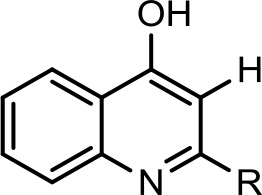	C_5:0_−**C**_5_**H**_11_	214	2	216	(+) 198, 194, 186, 172, 159, 146.	(+) 198, 184, 172, 159	Lepine et al., 2004
		C_6:0_−**C**_6_**H**_13_	228	1	230	(+) 212, 202, 194, 186, 172, 159, 146.		Deziel et al., 2004
		C_7:1_−**C**_7_**H**_13_	240	2	242	(+) 224, 213, 200, 194, 186, 185, 184, 172, 159, 146. (−) 198, 184, 172, 170, 158, 157, 144, 143.	(+) 198, 184, 172, 159	Deziel et al., 2004; Lepine et al., 2004
		C_7:0_−**C**_7_**H**_15_	242	28	244	(+) 200, 188, 186, 172, 159, 146. (−) 228, 212, 198, 184, 170, 158, 157, 144, 143.	(+)186, 172, 159, 146.	Vial et al., 2008
		C_8:0_−**C**_8_**H**_17_	256	6	258	(+) 240, 224, 198, 188, 186, 172, 159, 146.		Deziel et al., 2004
		C_9:1_−**C**_9_**H**_17_	268	14	270	(+) 228, 185, 172, 159, 146.	(+) 184, 172, 159	Lepine et al., 2004
		C_9:0_−**C**_9_**H**_19_	270	24	272	(+) 186, 172, 159, 146. (−) 184, 170, 158, 157, 144.	(+) 184, 172, 159	Lepine et al., 2004
		C_11:1_−**C**_11_**H**_21_	296	15	298	(+) 298, 270, 256, 242, 228, 214, 200, 186, 172, 160, 159.	(+) 160, 174	Vial et al., 2008
		C_13:1_−**C**_13_**H**_25_	324	4	326	(+) 308, 293, 284, 270, 256, 242, 228, 214, 200, 186, 172, 159. (−) 306, 296, 290, 286, 280, 260, 246, 244, 242, 223, 205, 184, 170, 158.		Lepine et al., 2004
2	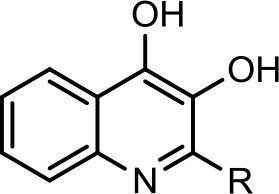	**C**_7_**H**_15_	258	100	260	(+) 242, 186, 175, 172, 162, 159. (−) 241, 240, 230, 214, 187, 173, 172, 159, 144.	(+) 188, 175	Lepine et al., 2004
3	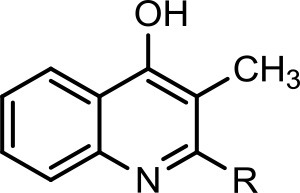	**C**_8_**H**_17_	268	14	270	(+) 228, 200, 186, 185, 184, 173, 172, 160, 159. (−) 226, 198, 184, 173, 170, 158, 157, 144, 143.	(+) 186, 173	Deziel et al., 2004; Vial et al., 2008
4	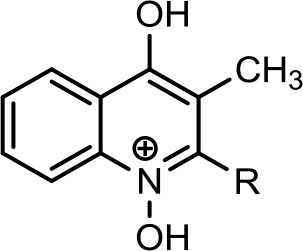	C_8:1_−**C**_8_**H**_15_	284	11	286	(+) 286, 268, 258, 240, 226, 216, 212, 202, 198, 188, 186, 184, 174, 172, 162, 160, 159, 146, 132.		Vial et al., 2008
		C_8:0_−**C**_8_**H**_17_	286	25	288	(+) 272, 186, 172, 159, 146. (−) 269, 268, 258, 242, 186, 174, 170, 159, 158, 157, 144.	(+) 188, 186, 172, 159, 144	Lepine et al., 2004; Vial et al., 2008
5	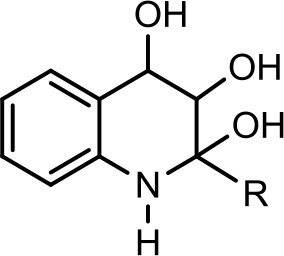	**C**_7_**H**_15_	278	12	280	(−) 278 → 242 → 170, 158, 157, 144		Current study
6	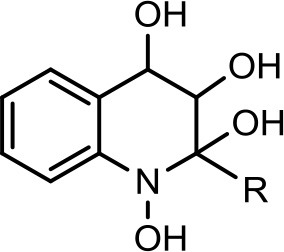	**C**_7_**H**_15_	294	30	296	(−) 279, 266, 258, 249, 248, 236, 223, 208, 196, 194, 184, 183, 170, 158.		Current study
		C_8:1_−**C**_8_**H**_15_	306	19	308	(−) 306 → 270 → 252, 236, 226, 198, 183, 170, 158, 157, 144.		Current study
7	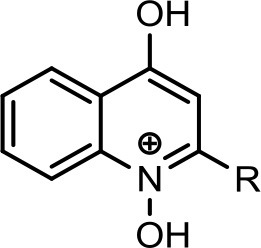	C_12:1_−**C**_12_**H**_23_	340	7	342	(−) 322, 312, 297, 291, 277, 260, 198, 184, 183, 170, 144.		Lepine et al., 2004

* MS/MS verified result are 2% normalized [Only those fragments (m/z) are mentioned whose abundance is >2%].

** *Percent relative abundance of peaks with respect to the base peak at m/z 258 (derived from negative ionization mode, Figure 2, Table-entry 2)*.

**Figure 3:**
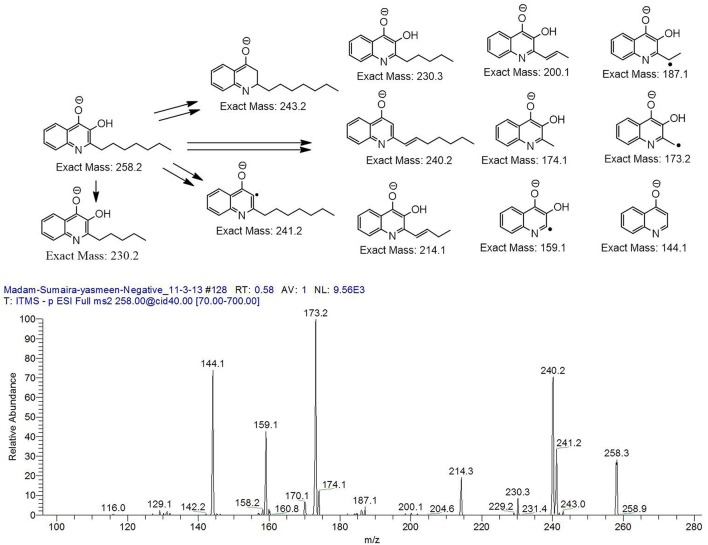
Profiling of the fragmentation data generated through the MS/MS of *m*/*z* 258 using CID (energy 40.0) at negative ion mode.

The bacterium demonstrated the capability to produce large variety of HAQs. Molecular ion peaks of 17 HAQs, representing seven analogous series, were identified (Table 2). These HAQs were classified into various groups on the basis of hydrogen, alkyl and hydroxyl groups at the two and three positions of heterocyclic ring, as well as *N*-oxide group at the position of quinoline nitrogen (Serial No. 1–7, Table 2). Serial No. 1 HAQs represent the eight analogs, having hydrogen at 3-position with *m/z* [M+H]^+^ values 216, 230, 242, 244, 258, 270, 298, and 326. These HAQs are relatively simpler 4-hydroxy-2-alkylquinolines, which only varied from each other on the basis of saturated or unsaturated alkyl side chain length. The molecular ions peaks [M+H]^+^ and [M-H]^−^ of these HAQs were further analyzed by collision induced dissociation (CID) and compared with literature data. In Serial No. 2 (Table 2), an HAQ having *m/z* at 258 [M-H]^−^, exhibited the highest peak intensity in full scan MS negative ion mode (Figure 3), representing a 3,4-dihydroxy-2-heptylquinoline (HHAQ), in which hydrogen at 3-position is substituted with hydroxyl group (Deziel et al., 2004). The structure of this molecule has been thoroughly investigated through collision induced dissociation at positive and negative ion modes to unambiguously profile the fragmentation data. Assigning the three daughter peaks can confirm the structure of HHAQ (Table 2, Figure 3).

HAQ representing *m/z* at 270 [M+H]^+^ & 268 [M-H]^−^ (Table 2, Serial No. 3) exhibited the structure of 4-hydroxy-3-methyl-2-alkylquinolines (HMAQ). Fragmentation of the parent peaks through CID produced the daughter ions, which correlated well with the proposed structure. Two analogs of 4-hydroxy-3-methyl-2-alkylquinolines *N*-oxide (HMAQ *N*-oxide) *m/z* at 286 and 288 [M+H]^+^, were also spotted (Table 2, Serial No. 4), exhibiting octaene and octane side chains, respectively.

**Figure 4:**
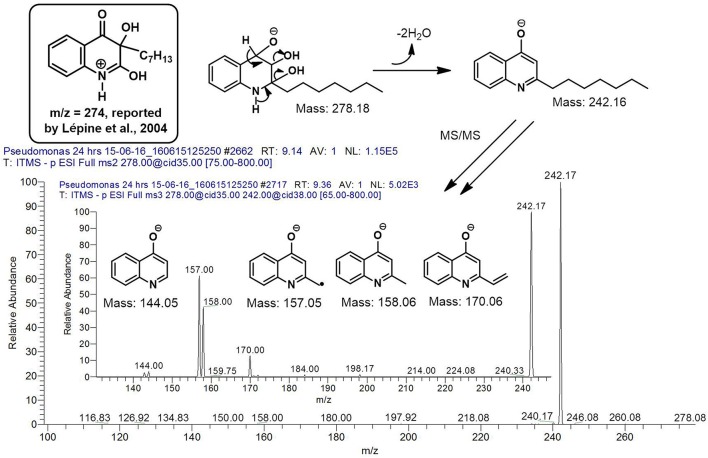
Putative structures of the fragment ion produced by CID of the *m*/*z* 278 [M-H]^−^ of Series 5 compound.

**Figure 5:**
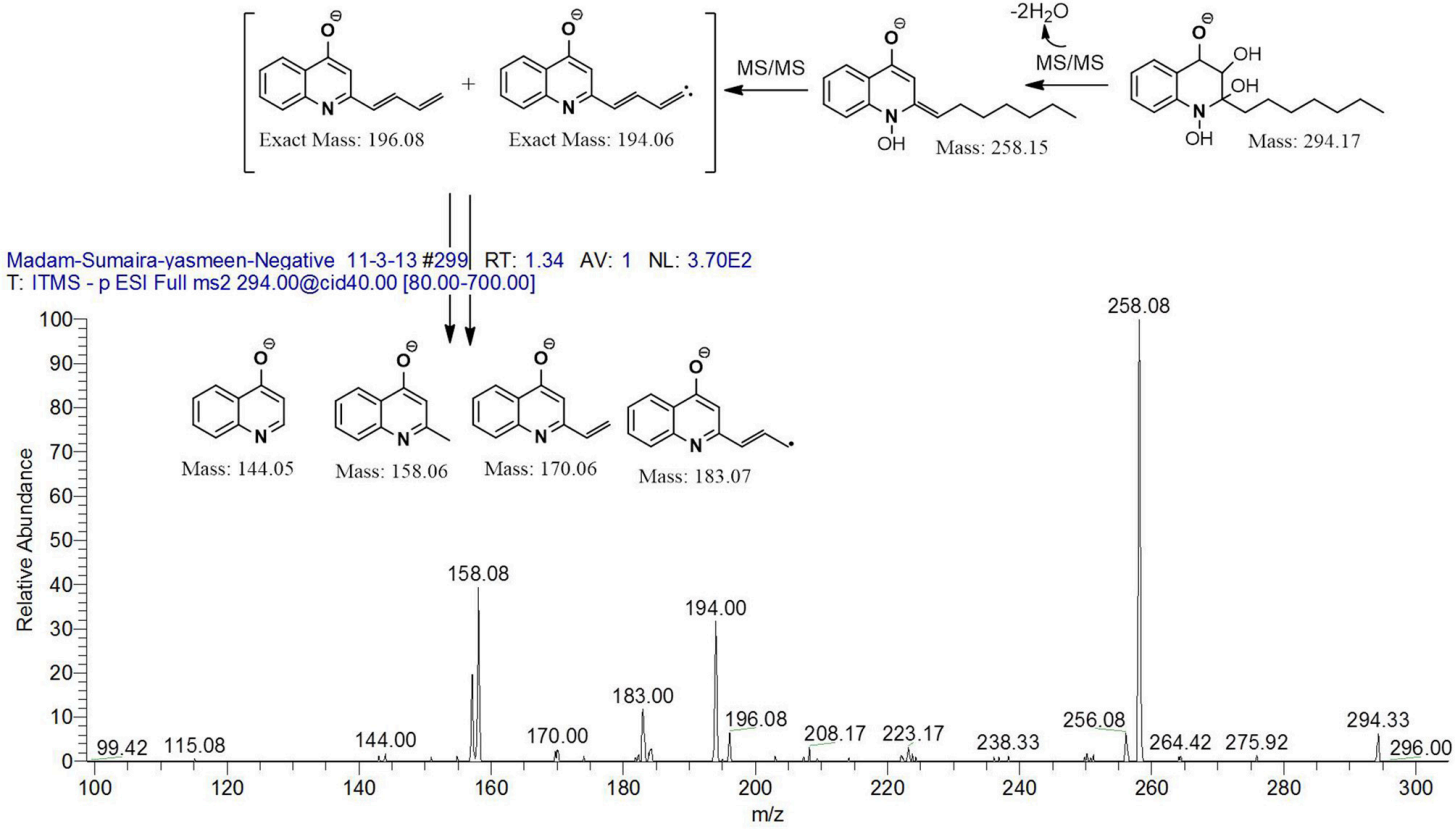
Putative structures of the fragment ion produced by CID of the *m*/*z* 294 [M-H]^−^ of Series 6 compounds.

However, to our surprise, a poly-hydroxy HAQ analog having *m/z* at 278 [M-H]^−^, demonstrating an interesting putative structure of 2,3,4-trihydroxy-2-alkylquinoline (HHHAQ), was identified at negative ion mode (Table 2, Serial No. 5). On collision induced dissociation, this molecule spontaneously lost two moles of water, leaving a stable ion species at *m/z* 242 (4-hydroxy-2-alkylquinoline), which on further fragmentation yielded the expected daughter ions at *m/z* values of 198, 184, 170, 158, 157, and 144 (Figure 4). This hydroxylation trend was further enhanced in Serial No. 6 compounds (Table 2), whose putative structures were assigned as 2,3,4-trihydroxy-2-alkylquinolines *N*-oxide (HHHAQ *N*-oxide). Two analogs in this tetrahydroxyl series were identified with *m/z* 294 and 306 [M-H]^−^, representing 2-heptyl and 2-octaenyl side chains, respectively. Collision induced dissociation of *m/z* 294 yielded the major daughter ion at *m/z* 258 after losing 2 moles of H_2_O (Figure 5). The other fragmentation products having *m/z* values of 196, 194, 183, 170, 158, and 144 were also structurally assigned, which supported the putative structure of *m/z* 294. Similarly, collision induced dissociation of *m/z* 306 ion, produced a stable daughter ion at *m/z* 270 after the loss of 2 moles of water, which on further fragmentation yielded daughter ions at *m/z* values of 252, 242, 181, 170, 158, 157, and 144 (Figure S4). Finally, 4-hydroxy-2-alkylquinoline *N*-oxide (HAQ *N*-oxide) representing *m*/*z* at 340 [M-H]^−^ (Table 2, Serial No. 7) was detected. Fragmentation of *m/z* 340 through CID demonstrated the daughter ions at *m/z* values of 322, 312, 297, 291, 277, 260, 198, 184, 183, 170, and 144, which correlated with the expected fragments.

The supernatant of strain BRp3 harvested at different time intervals showed Xoo suppression in plate diffusion assay with an inhibition zone of 8–15 mm. The maximum antibacterial activity against Xoo was observed for the supernatant harvested after 24–48 h growth of bacterial culture (Figure S7).

### *In planta* evaluation for the suppression of BLB

#### Net house experiment

The bacterial strain BRp3 was studied for *in vivo* suppression of BLB in a pot experiment under net house conditions. Bacterial inoculation resulted in significant disease suppression shown by percent diseased leaf area (3.7% DLA) compared to that of the infected control (37.2%) against Xoo. Besides reducing the disease incidence, strain BRp3 improved the plant dry weight as compared to control plants without inoculation.

**Figure 6:**
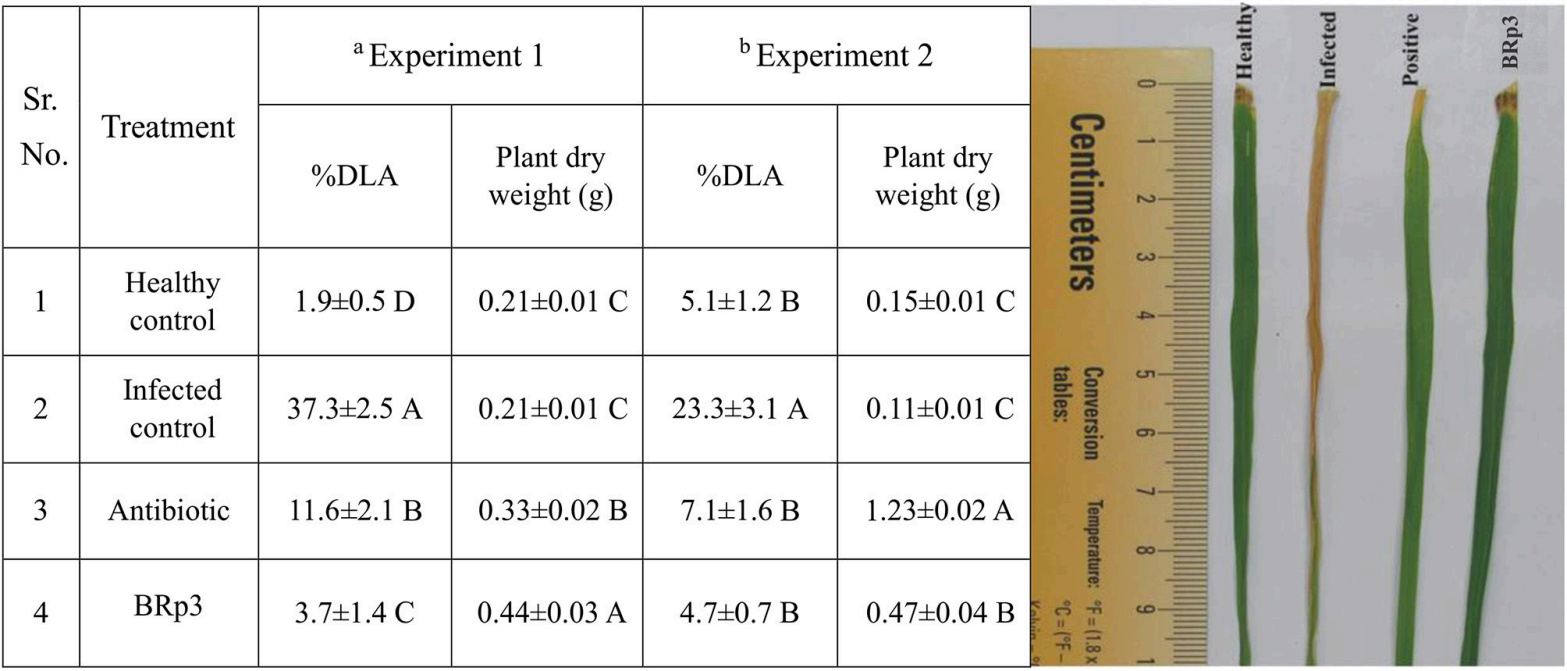
Effects of rice rhizosphere associated *Pseudomonas aeruginosa* BRp3 for suppression of bacterial leaf blight (BLB) in pot experiments under net house conditions. Antagonistic bacteria i.e., BRp3 was applied both as seed treatment and foliar spray 1 day before clip inoculation of Xoo pathogen. Antibiotic i.e., Streptocyclin @ 5 mg/ pot was sprayed 1 day before clip inoculation (Positive control). Means are an average of four biological replicates and there were 15 plants per replicate. Means followed by the same letter differ non-significantly at *p* = 0.01 according to DMRT. ^a^Experiment was conducted in small pots and ^b^experiment was conducted in large earthen pots. Different letters show statistical significance of treatments while similar letters show non-significant differences.

Another experiment conducted in large earthen pots showed that the effect of strain was statistically significant for disease suppression and growth promotion compared to infected and healthy control plants. DLA (%) of plants inoculated with BRp3 was 4.7% as compared to infected (23.3%) and streptocyclin treated plants (7.1%; Figure 6).

#### Induction of defense related enzymes

**Figure 7:**
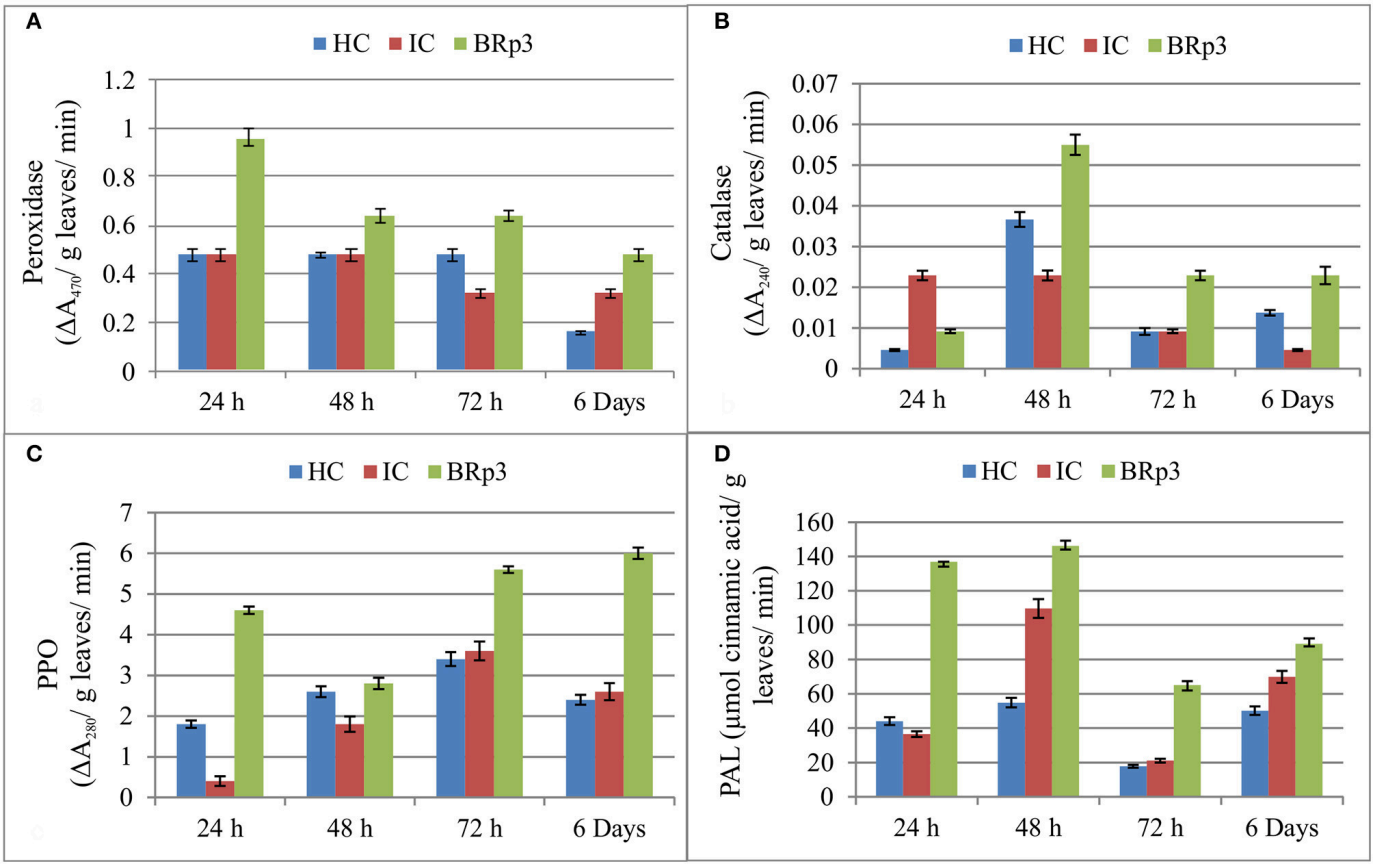
Induction of defense related enzymes **(A)** Peroxidase **(B)** Catalase **(C)** Polyphenol oxidase (PPO) and **(D)** Phenylalanine ammonia lyase (PAL) in rice plants inoculated with *Pseudomonas aeruginosa* BRp3 and challenge inoculated with bacterial leaf blight causing pathogen. Healthy control (HC): Leaves of plants clip inoculated with distilled water, Infected control (IC): Leaves clip inoculated with BLB pathogen i.e., *Xanthomonas oryzae* pv. *oryzae* (Xoo). Forty leaves/ treatment were clip inoculated. Bars show standard deviation of four biological replicates and each replicate has 15 plants per replicate.

The net house experiment to study the accumulation of defense related enzymes showed that upon Xoo clip inoculation of rice plants inoculated with *P. aeruginosa* strain BRp3, higher activities of defense related enzymes were observed at 24 and 48 h post-inoculation. Peroxidase was observed to be maximum as compared to healthy and infected control after 24 h of Xoo inoculation. The higher activity of Catalase was observed after 48 h of Xoo inoculation. The inoculation of BRp3 resulted in higher induction of Poly Phenol Oxidase (PPO) activity at 24, 72, and 144 h post-inoculation. Maximum activity of PAL was observed after 24 and 48 h of Xoo inoculation (Figure 7).

#### Field experiment

**Figure 8:**
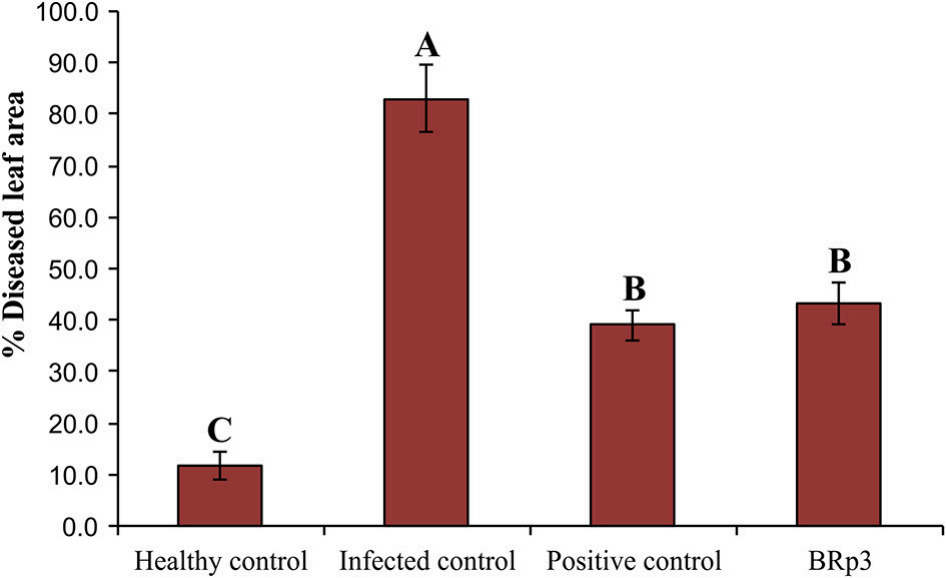
Evaluation of *Pseudomonas aeruginosa* BRp3 for suppression of bacterial leaf blight (BLB) under field conditions. Healthy control: Leaves of plants clip inoculated with distilled water, Infected control: Leaves clip inoculated with prevalent BLB pathogen i.e., *Xanthomonas oryzae* pv. o*ryzae* (Xoo) strain isolated in the present study; Positive control: Leaves clip inoculated with BLB pathogen and plants sprayed with streptocyclin and *Pseudomonas* sp. BRp3: Leaves clip inoculated with BLB pathogen and plants sprayed with liquid culture of strain BRp3. Sixty leaves/ treatment were clip inoculated. Four plants per replicate were sown in 1 m row. Suppression of BLB was measured in terms of reduction in the mean bacterial blight lesion length on treated leaves compared to those of non-inoculated control. Bars show standard deviation of three biological replicates. Means followed by the same letter differ non-significantly at *p* = 0.05 according to DMRT.

**Figure 9:**
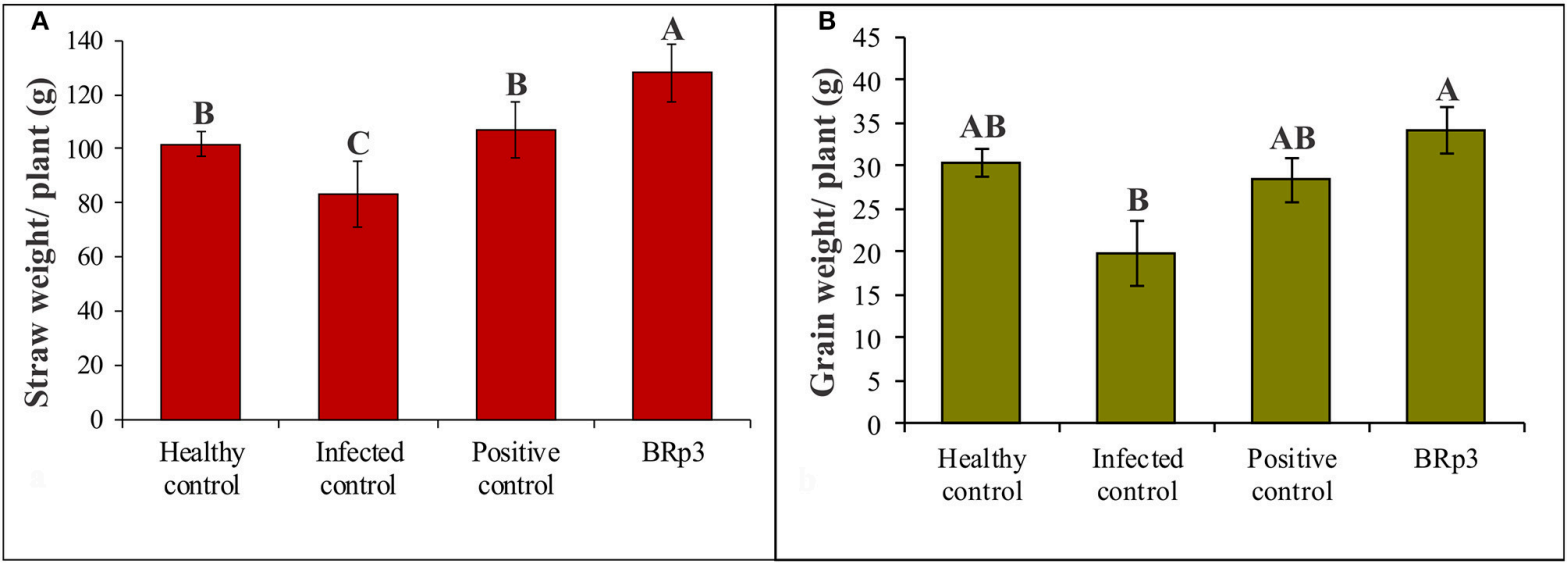
Effects of *Pseudomonas aeruginosa* BRp3 on yield parameters of rice in the presence of Xoo pathogen **(A)** straw weight and **(B)** grain weight. Four plants per replicate were sown in 1 m row; Bars show standard deviation of three biological replicates. Means followed by the same letter differ non-significantly at *p* = 0.05 according to DMRT.

Assessment of antagonistic strain BRp3 under field conditions was done at booting stage of rice variety Super Basmati. A prevalent virulent strain Xoo7 isolated in the present study was used for clip inoculation at booting stage. Application of *P*. *aeruginosa* strain BRp3 significantly reduced DLA i.e., 43% as compared with infected control i.e., 83%. Maximum reduction in disease severity was recorded by foliar application of streptocyclin with 39% DLA but it was non-significantly different from that of the strain BRp3 (Figure 8). The lesion length was 6.2–8.6, 5.6–9.9, and 8.5–17.9 cm in treatments of BRp3, streptocyclin and infected control plants, respectively. BRp3 inoculated rice plants showed a significant increase in straw yield. Grain weight per plant was maximum with the strain BRp3 i.e., 34.2 g plant^−1^ followed by healthy and streptocyclin treated plants. Minimum grain yield (19.8 g plant^−1^) was observed for infected control plants (Figure 9).

### Field evaluation for yield increase of rice

**Figure 10:**
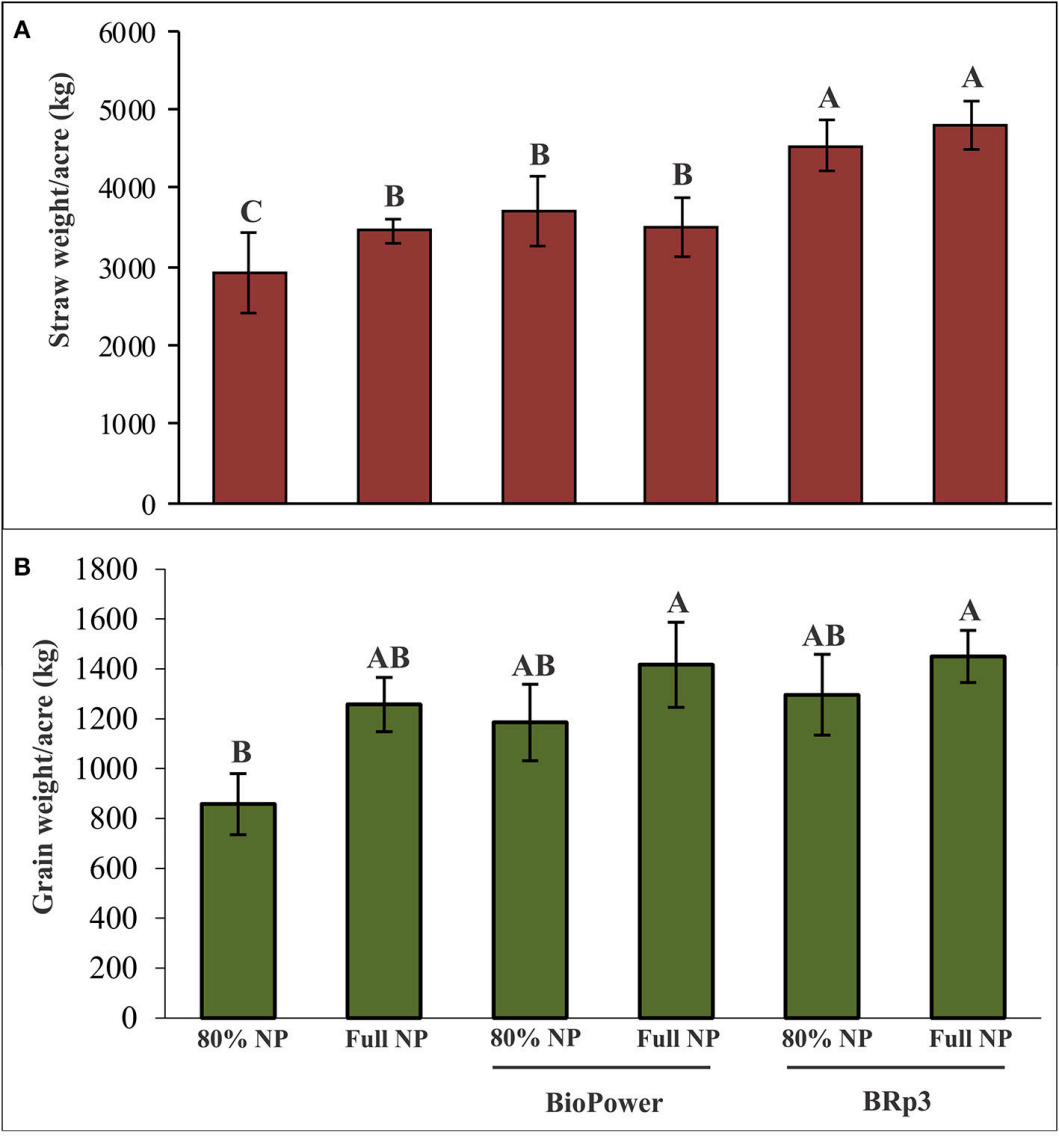
Effects of *Pseudomonas aeruginosa* BRp3 on yield parameters of rice variety *Super Basmati* in a field experiment in the absence of Xoo pathogen. **(A)** Straw weight and **(B)** grain weight. Un-inoculated with 80% of the recommended doses of nitrogen (N) and Phosphorus (P) fertilizers. Un-inoculated with full dose of the recommended N and P fertilizers. “*BioPower*” a consortium of bacterial strains is a commercial biofertilizer product of NIBGE. Bars show standard deviation of four biological replicates. Means followed by the same letter differ non-significantly at *p* = 0.05 according to DMRT. Plot size was 28 m^2^.

A field experiment was conducted to evaluate the inoculation effect of strain BRp3 on growth and yield of rice variety Super Basmati with different levels of fertilization. Application of the strain BRp3 either with 80% of the recommended doses or at full recommended doses of N and P, significantly increased the growth parameters in rice compared to respective non-inoculated control plants. The inoculation with the strain BRp3 showed 55% increase in straw and 51% in grain compared to non-inoculated control (with 80% N and P; Figure 10).

### Rhizosphere colonization studies

**Figure 11:**
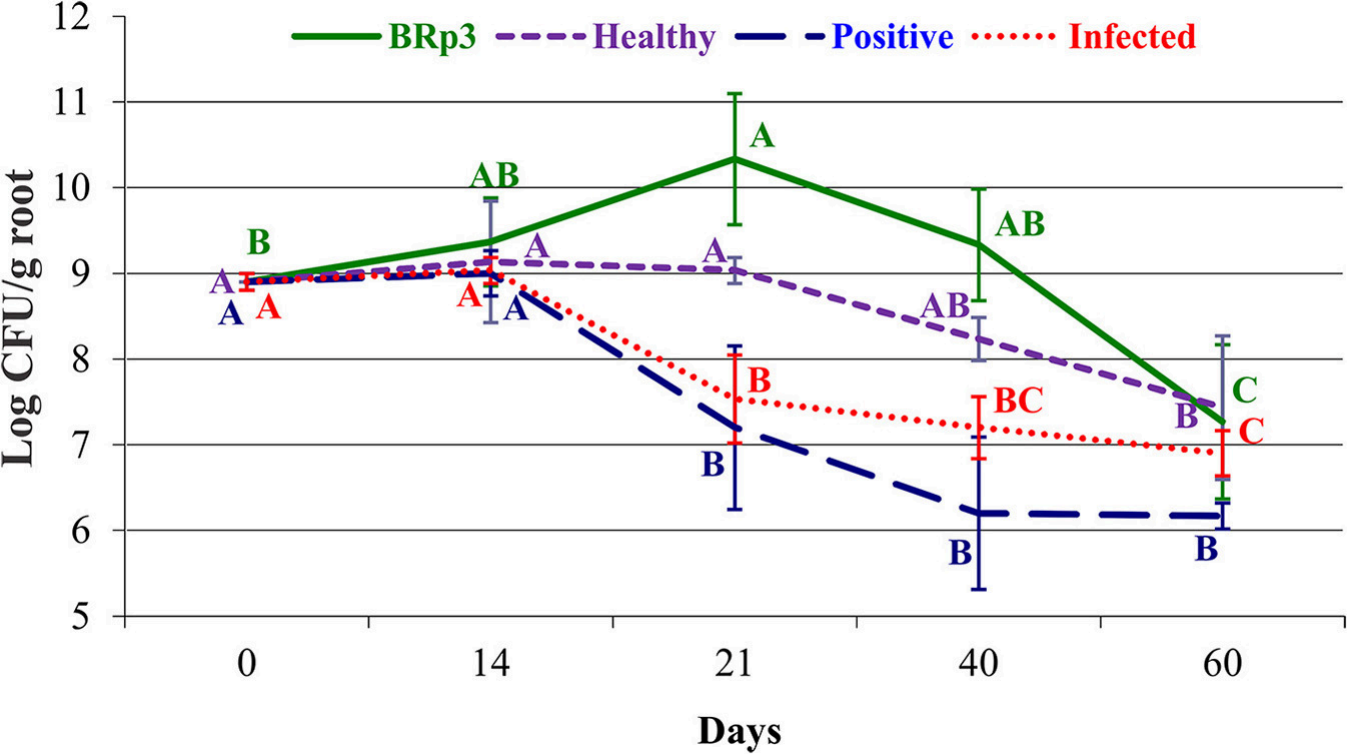
Rice root colonization in response to different treatments in a field experiment. *Pseudomonas aeruginosa* BRp3 was applied both as seed treatment and foliar spray 1 day before clip inoculation of rice bacterial blight pathogen. Foliar spray of antibiotic streptocyclin was used as positive control while non-inoculated plants with and without pathogen, were used as infected and healthy controls, respectively. Bars represent the standard deviation of three biological replicates. Four plants per replicate were sown in 1 m row and three root samples from each replicate were collected. Means followed by the same letter differ non-significantly at *p* = 0.05 according to DMRT. CFU, Colony forming units represent total viable count.

**Figure 12:**
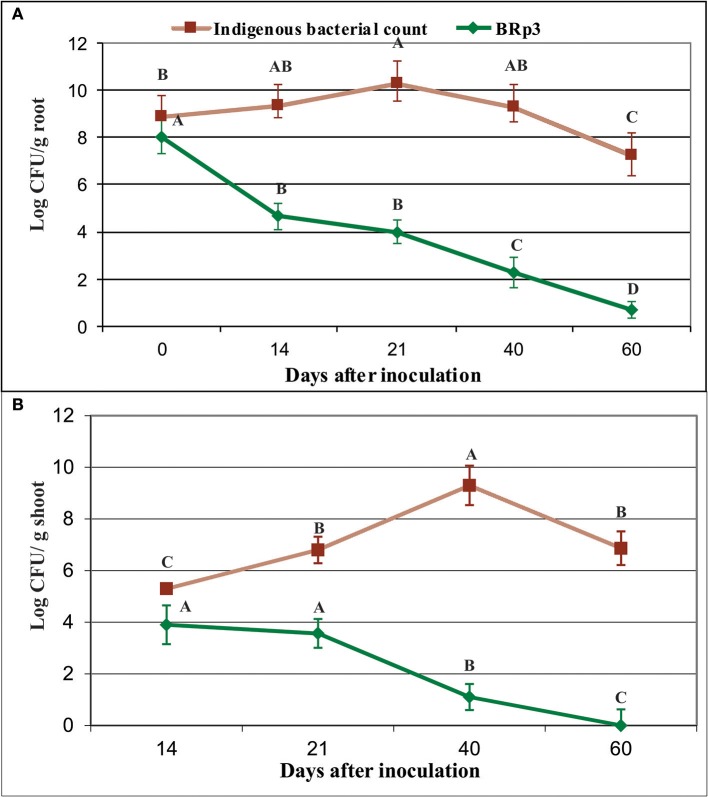
Survival of *Pseudomonas aeruginosa* BRp3 on the roots **(A)** and shoots **(B)** of field-grown rice plants. Enumeration of bacterial population was determined by viable count method. Root and shoot samples were collected from field-grown rice plants. Four plants per replicate were sown in 1 m row and three root/shoot samples from each replicate were collected. Bars represent the standard deviation. Means followed by the same letter differ non-significantly at *p* = 0.05 according to DMRT.CFU, Colony forming units.

Colonization studies under field conditions showed that total culturable indigenous bacterial population on rice roots as log_10_ 8.9–7.3 CFU g^−1^ root (Figure 11, Figure S8). The survival level of BRp3 however, declined gradually after seed treatment from log_10_ 8 to 0.7 CFU g^−1^ root (Figure 12A). Total culturable indigenous bacterial counts on rice shoots were log_10_ 5.3–6.9 CFU g^−1^ shoot up to 40 days after sowing. The bacterial counts of strain BRp3 was log_10_ 3.9–1.1 CFU g^−1^ shoot when enumerated DPI while no colony of strain BRp3 was detected from surface-sterilized shoots of rice plant at 60 DPI (Figure 12B). Antibiotic resistance pattern (Table S3), *in vitro* suppression of Xoo growth (20 mm inhibition zone) and the other plant growth promoting traits like P solubilization (94 ± 3 μg mL^−1^), production of IAA (28 ± 2 μg mL^−1^), and siderophores 12 ± 2 mg L^−1^) of the inoculated bacterium were found comparable to those observed earlier for its pure culture.

Counts of total culturable bacteria on rice roots increased with plant growth. In the rhizosphere, the higher number (log_10_10.3 CFU g^−1^ root) of bacteria was found on BRp3-inoculated plants, followed by healthy control plants (log_10_ 9 CFU g^−1^root) determined at 21 DPI. A decreasing pattern in the counts was more for infected (inoculated with Xoo only) and positive controls (streptocyclin) as compared to that of the strain BRp3 inoculated plots.

**Figure 13:**
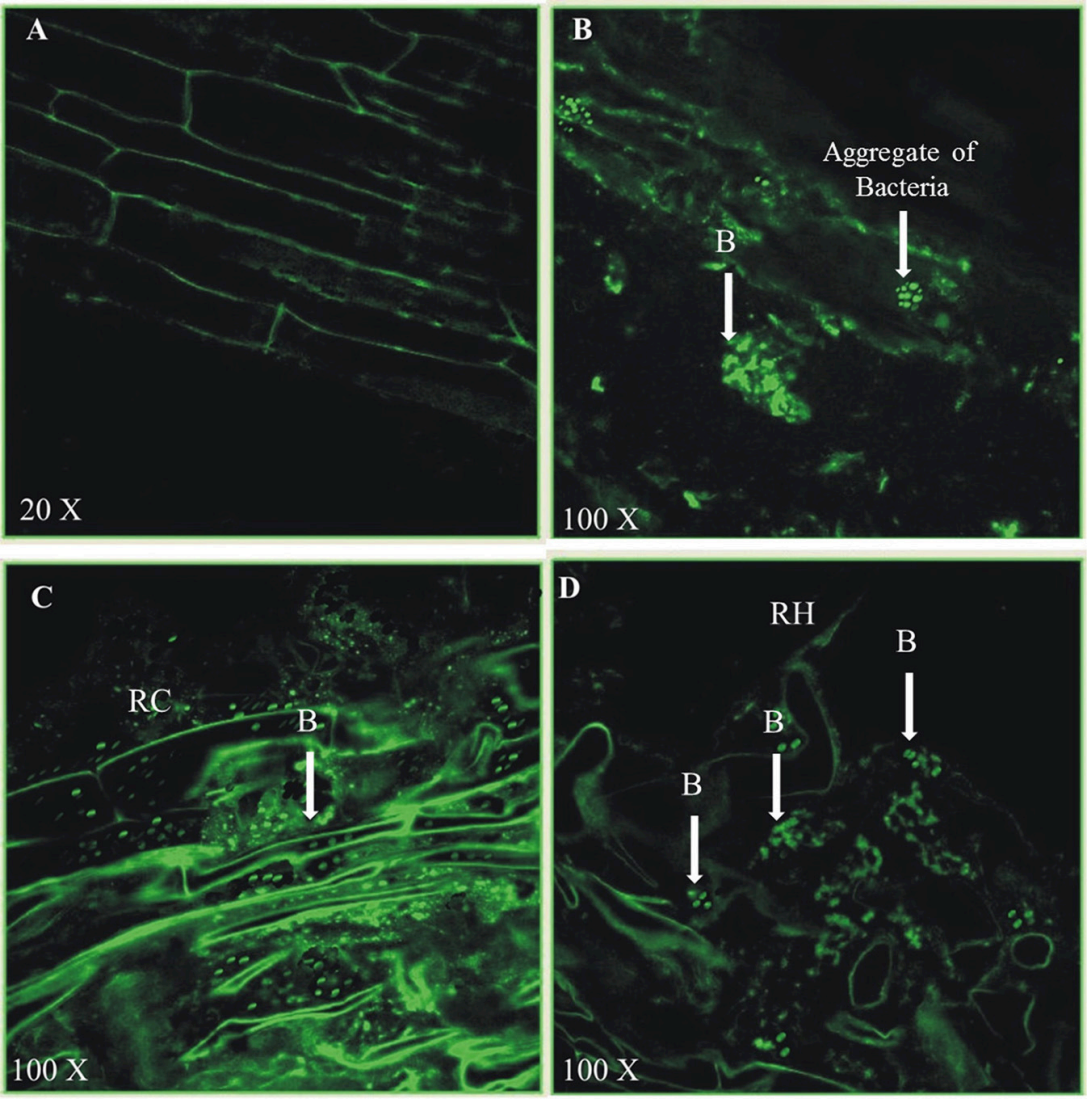
Colonization of 21-days old rice roots by *Pseudomonas aeruginosa* BRp3 studied by fluorescence antibodies staining and CLSM. **(A–D)** FITC-Immunofluorescence images by confocal laser scanning microscopy (CLSM) of whole root after staining by fluorescent antibody (FA) technique. Un-inoculated control **(A)**, Rice roots inoculated with *Pseudomonas aeruginosa* BRp3 **(B,C)** grown in sterile sand under net house conditions and **(D)** under field conditions. B, Bacteria; RC, Root cell; RH, Root hair.

Colonization of *P. aeruginosa* BRp3 on rice roots was studied with the help of immunofluorescence (IF) assay and confocal laser scanning microscopy (CLSM). The primary antiserum raised against strain BRp3 was found to be strain specific (Figure S9). Strain BRp3 was found to colonize all over the rice roots at 100X resolution. No fluorescence was observed on the roots of un-inoculated plants (Figure 13A). Colonization was observed in intercellular spaces and in micro-colonies as well (Figures 13B–D). However, the number of BRp3 cells detected by confocal microscopy decreased gradually after 21 days of seed treatment.

## Discussion

Plant-associated beneficial bacteria are important growth promoters or biocontrol agents in modern agriculture where eco-friendly and sustainable approaches are growing and have more acceptance than ever before. Rice is grown on large area of Pakistan where a lot of chemical fertilizers as well as pesticides are applied. This study was carried out to evaluate the potential for rhizobacterial inoculum to promote growth and to control disease in rice.

The pathogenicity of *X. oryzae* strains to rice confirmed their identity as causal pathogen of BLB. Xoo1 and Xoo2 were used for further studies due to their aggressive behavior. *In vitro* plate assay for screening the rhizobacterial isolates (512) for inhibition of growth of Xoo led to the selection of *P. aeruginosa* BRp3 for further studies. In a previous study, only LB medium was used for isolation of antagonistic bacteria and the frequency of antagonistic bacteria among the total isolated bacterial population was low (Yasmin et al., 2016). A relatively higher number of antagonistic bacteria was obtained in the present study when different growth media (Nutrient Agar, King's B, and Gould's S1) were used for isolation of bacterial populations. As the biocontrol agents with broad spectrum antagonism are found to be more effective against phytopathogens in rhizosphere, therefore, the antagonistic bacteria isolated in the present study were also screened against different phytopathogens, other than the target pathogen (Table S1). Several rice varieties are infected by various fungal pathogens, including *Fusarium* spp. that cause bakanae disease in rice (Desjardins et al., 2000). The suppression of *in vitro* growth of the *Fusarium* spp. by the antagonistic bacteria showed its potential use as an inoculant for biocontrol of these fungal pathogens.

Phosphate solubilizing and IAA producing microbes are a vital fraction of the microbes that improve the development and growth of their host plant. Vassilev et al. (2006) reported that the solubilization of insoluble phosphates by microbial activity usually induce the secretion of certain metabolites mainly siderophores, lytic enzymes and phytohormones that are involved in the control of phytopathogens. The intrinsic resistance of strain BRp3 against different antibiotics may aid survival in the rhizosphere whenever used as a biological control agent (Dobereiner and Baldani, 1997). In our previous study, siderophore production and P-solubilization were found to be involved in growth promoting activities of antagonistic *P. aeruginosa* Rh323 (Yasmin et al., 2016) whereas the present study suggests that P solubilization accompanied with the production of IAA, siderophore and ACC may contribute to the growth promoting activities of BRp3, even in the presence of Xoo. ACC deaminase activity of this strain may help to lower the level of ACC caused by ethylene under stress conditions and protect the host plant. Bacterial ACC-deaminase is known to regulate plant growth under biotic and abiotic stress conditions (Singh and Jha, 2016).

It is necessary to understand the mechanisms involved in the suppression of pathogens by the application of biocontrol agents for effective disease management. The bio-antagonistic bacterium used in the present study was characterized for different biocontrol determinants i.e., HCN, chitinases, proteases, starch hydrolysis and siderophores production. Strain BRp3 produced volatile compounds such as HCN which is a known suppresser of phytopathogens (Brimecombe et al., 2001). Recent literature reported that cyanide producing bacteria can be considered as effective biocontrol agents because cyanide production by the bacterial strains induces resistance in the plant (Devi and Kothamasi, 2009; Spence et al., 2014). Gandhi et al. (2009) reported the production of an antifungal protease by rice rhizosphere associated *Chryseobacterium aquaticum* PUPC1 and its effects on the mycelial growth, germination of spores, and sclerotia of phytopathogenic fungi. It has been reported that chitinase can function in defense against many fungal pathogens and also correlated with induced resistance (Perez et al., 2002). Starch hydrolysing ability of the strains indicated their capability to produce amylase and to use a complex carbon source, which aids in the defensive mechanisms of bacterial strains (Marten et al., 2000).

There are many reports that highlight the importance of secondary metabolites in the biocontrol of plant pathogens (Heeb et al., 2011; Khare and Arora, 2011; Jayaseelan et al., 2014). *P. aeruginosa* BRp3 has the capability to produce a variety of metabolites, which include siderophores (1-hydroxy-phenazine, pyocyanin and pyochellin), rhamnolipids and a series of already characterized 4-hydroxy-2-alkylquinolines (HAQs) as well as novel 2,3,4-trihydroxy-2-alkylquinolines and 1,2,3,4-tetrahydroxy-2-alkylquinolines. Mass spectrometric analysis has confirmed their structures (Figures 1, 2, Table 2).

Siderophores have also been reported to induce systemic acquired resistance (Trivedi et al., 2008; Sulochana et al., 2014). *P. aeruginosa* BRp3 produces 1-hydroxy-phenazine, pyocyanin (Figure 1) and pyochelin (*m/z* 325, Figure 2). Phenazine produced by non-pathogenic strain of *P. aeruginosa* has been documented as an antifungal metabolite on the basis of NMR and MS analyses (Kumar et al., 2011). Pyochelin produced by *P. aeruginosa* showed antagonistic activity against *Botrytis cinerea*, the phytopathogen of groundnut (Khare and Arora, 2011). Purified pyocyanin produced by *P. aeruginosa* TO3 was reported for its inhibitory effect against *Macrophomina phaseolina* in tomato (Audenaert et al., 2002).

Rhamnolipids are widely reported to be produced by various microbes including the *Pseudomonas* spp. (Chong and Li, 2017). *P. aeruginosa* BRp3 demonstrated the production of mono- and di-rhamnolipids (ranged from *m/z* 500 to 650, Figure 2). LCMS analysis confirmed that the rhamnolipids produced by *P. aeruginosa* DR1 inhibited the growth of different plant pathogens like *F. oxysporum, Sclerotium rolfsii, Phytophthora nicotianae, and M. phaseolina* (Reddy et al., 2016).

*P. aeruginosa* BRp3 is capable of producing a large repertoire of HAQ and HAQ-related compounds in addition to the siderophores and rhamnolipids. Mass spectrometric analysis of cell free extract revealed the presence of nine analogs of 4-hydroxy-2-alkyl HAQs, having saturated and unsaturated alkyl carbon chains, varied from C_5_ to C_13_ chain length, exhibiting *m/z* values of 216–326 [M+H]^+^ (Table 2, Serial No. 1). Their fragmentation data, can confirm their structures, which is well correlated with previously reported literature data (Deziel et al., 2004; Lepine et al., 2004; Vial et al., 2008). Only one analog of the structure representing 3,4-dihydroxy-2-heptyl (HHAQ) was identified (Figure 3). HHAQ has demonstrated the highest peak intensity at *m/z* 258 (Figure 2), this compound also termed as “*Pseudomonas* Quinolone Signal” (PQS) and is involved in the mechanisms of cellular communication, to detect the pseudomonas cell density (Deziel et al., 2004).

Interestingly, putative polyhydroxy HAQ analogs, i.e., a 2,3,4-trihydroxy-2-alkylquinoline *m/z* 278 [M-H]^−^ and two analogs of 2,3,4-trihydroxy-2-alkylquinoline *N*-oxideat *m/z* 294 and 306 [M-H]^−^, respectively, were found to be present in crude extract (Series 5 and 6 compounds, Table 2). The fragmentation data correlated with the putative structures of these polyhydroxy HAQs (Figures 4, 5). Budzikiewicz and Kesselmeier (1979) and Neuenhaus et al. (1979) reported the putative 3-alkyl-3-hydroxy-2, 4-dioxo-1, 2, 3, 4-tetrahydroquinoline derivatives in *Pseudomonas*. To support the theme of 3-alkyl substitution, they further synthesized these compounds (Figure S5). Similarly, in a detailed study conducted by Lepine et al. (2004) on metabolic profiling of HAQs from *Pseudomonas*, polyhydroxy HAQs were spotted but the alkyl side chain was reported on 3-position. However, to the best of our knowledge the polyhydroxy HAQs representing alkyl groups at 2-position of the heterocyclic rings, have not been reported in *Pseudomonas* species before this study. To support our theory of alkyl substitution at 2-position, the proposed 2,3,4-trihydroxy-2-alkylquinoline at *m/z* 278 was fragmented through CID that generated predominant daughter ion at *m/z* 242, which on further fragmentation yielded similar finger prints of ions as produced by 4-hydroxy-2-heptylquinolines (Table 2, Serial 1; Figure S6). Finally, a 4-hydroxy-2-dodecenylquinoline *N*-oxide analog at *m/z* 340 [M-H]^−^ was identified, whose MS/MS data was correlated with the proposed structure (Table 2, Serial No. 7). The production of this HAQ by *Pseudomonas*, has also been reported by Lepine et al. (2004). *Pseudomonas aeruginosa* and other related bacteria produced 2-alkyl-4(1H)-quinolones, which exhibited antimicrobial activity (Heeb et al., 2011).

This work demonstrates that *P. aeruginosa* BRp3 is capable of producing a large repertoire of HAQ and HAQ-related compounds in addition to the siderophores and rhamnolipids. Identification of polyhydroxy HAQs may provide insight about the biosynthetic pathway of these interesting compounds. These HAQs along with siderophores in the presence of rhamnolipids (as emulsifying and bacterial cell membrane disrupting agents), may be collectively responsible for the profound antibacterial activity of BRp3 against Xoo. Further studies are needed to establish the individual role of each detected secondary metabolite entity as an active biocontrol agent against BLB pathogen. But the challenge to such study is to get these metabolites in measurable quantity in their purified form. Since these metabolites are being produced in nano grams concentration, the optimization of their production through fermentation would have to be optimized prior to their purification process. Secondly, their purification is challenging, owing to the slight variation in their structures, polarity, hydrophobicity etc. (especially the HAQs), and a routine silica column chromatography was failed to purify them. Although, Naureen et al. (2017) have successfully purified the secondary metabolites (2-pentyl-4-quinolinecarboxylic acid and 1- methylcyclohexene) from *Lysinibacillus sphaericus* ZA9 but the chemistry of these metabolites is significantly different from the HAQs identified in the present study. A high-quality preparatory HPLC system may be required to purify various analogs of HAQs (Wang et al., 2011).

Due to the aforementioned technical challenges, obtaining the sufficient quantities of purified metabolites for plant assays looks difficult. However, crude supernatant from pure cultures of BRp3 were shown to suppress the BLB pathogen (Figure S7). The extracted supernatant of antagonistic bacterial culture has been reported for the control of phytopathogens during *in-planta* evaluation (Simonetti et al., 2012). The extracellular filtrates of biocontrol bacteria having different secondary metabolites such as cyclic lipopeptides were found to be responsible for their antifungal activity (Petatan-Sagahon et al., 2011). Few reports have documented the significant pathogen suppression by supernatants of fermentation without extraction (Garcia et al., 2014). The mechanistic suppression of BLB can be supported by the fact that rice plants inoculated with the strain BRp3 and challenge inoculated with BLB pathogen resulted in an increased activity of defense related enzymes in the host plant. In a previous study, accumulation of defense related enzymes was observed in rice plant up to 48 h after foliar application of antagonistic bacteria Rh323 (Yasmin et al., 2016). Plants inoculated with BRp3 exhibited an increase in the activity of POD, CAT, and PAL after 24 and 48 h post inoculation while, PPO activity was observed even after 72 h and up-to 6 days after challenge inoculation. The production of plant defensive enzymes i.e., POD, PPO, CAT, and PAL in response to inoculated PGPR bacteria is associated with ISR in plants against the pathogen (Saikia et al., 2006; Liang et al., 2011).

A better evaluation of plant growth promoting efficiency of the bacterial agents under net house conditions is one of the prerequisites for transferring a strategy from the laboratory into the field. *P. aeruginosa* BRp3 showed effective and consistent pathogen suppression in different pot experiments conducted under net house conditions with different strains of BLB pathogen as indicated by significantly reduced diseased leaf area as compared to the respective non-inoculated control (infected control) as well as compared to positive control of streptocyclin treated plants. Velusamy et al. (2006) reported 58.8 and 64.5% BLB suppression by a DAPG producing *P. fluorescens* PTB9 in net house and field experiments, respectively. Literature showed that the application of *P. fluorescens* Pf1 treatment effectively controlled BLB and the treatment was found to be more effective than the standard streptocyclin in controlling the disease as well as in increasing the yield (Vidhyasekaran et al., 2001).

Field experiments conducted in the presence of Xoo indicated that inoculation with *P. aeruginosa* BRp3 significantly reduced diseased leaf area compared to respective non-inoculated control (infected control) but non-significantly compared to positive control of streptocyclin treated plants. Strain BRp3 showing suppression of BLB equivalent to the positive control under field conditions can be considered as a promising biological control agent to suppress Xoo in rice. Root or seed inoculation of rice plants with this bacterium provided protection to the plant against BLB at early growth stages while its foliar spray at later growth stage or maximum tillering stage protected the rice plant from subsequent disease incidence. Various secondary metabolites produced by this strain along with HCN, may be responsible for the suppression of phytopathogens. The production of increased biomass by inoculated BRp3 may be induced by the production of IAA or siderophores. Different studies reported enhanced growth of root-shoot length of cucumber, lettuce, potato and tomato due to inoculated *Pseudomonas* strains (Weller, 2007). Khare and Arora (2010) reported the role of IAA produced by *P*. *aeruginosa* in the suppression of charcoal rot disease of chickpea. Different types of siderophores such as pesudobactin and pyoverdine produced by rhizospheric bacteria chelate to the available form of iron present in the soil and suppress the pathogens by reducing the availability of iron for the phytopathogen (Wensing et al., 2010; Sulochana et al., 2014). Notably, the number of rhizosphere-associated *Pseudomonas* species involved in yield enhancement (Combes et al., 2011) and/or reduction in plant diseases, are increasing (Couillerot et al., 2009; Beneduzi et al., 2012).

The inoculated bacteria BRp3 supplemented with 80% of the recommended doses of N and P significantly enhanced the grain and straw yield with 51 and 55% increase, respectively as compared to the respective control. The single inoculated strain BRp3 showed yield increase comparable to that of commercial biofertilizer (*BioPower*). Application of strain BRp3 either with 80% of the recommended doses or at full/ recommended doses of N and P, significantly improved the growth parameters in rice variety Super Basmati in comparison with control plants without inoculation provided with the same doses of N and P. *BioPower* treatment also significantly increased the grain weight and straw weight even with 80% of the recommended doses of nitrogen and phosphorus (Figure 10). The inoculation effect of BRp3 on straw and grain yield with different levels of fertilization indicated that the inoculated bacteria may contribute equal to that of the 80% of the recommended doses of N and P indicating that it may save 20% of urea and DAP fertilizers during crop growth season. These results suggest that the rhizobacteria-inoculants can be applied after further evaluation for nutrient management programs. Adesemoye et al. (2009) reported that supplementing 75% of the recommended fertilizer rate with inoculants produced plant growth, yield, and nutrient (N and P) uptake that were statistically equivalent to the full fertilizer rate without inoculants. The use of fertilizer at rates below the recommended dose, in the absence of bioinoculants, resulted in inconsistent effects on the plant with significant reduction in nutrient uptake and yield. In a previous study by Yasmin et al. (2016), inoculation of *Pseudomonas* spp. and *Serratia* sp. had a significant effect on rice growth under net house conditions and the grain yield was increased in the field too when used in mixed consortia but the effect of field inoculation was non-significant compared to the respective control. In the present study, BRp3 as a single inoculum, not only suppressed the pathogen under field conditions but also improved the rice yield significantly. The factors contributed for higher grain yield due to strain BRp3 as compared to the previously isolated antagonistic strains may be due to its higher potential for IAA and siderophores production. The siderophores produced were also evidenced by LCMS analysis.

Several studies have demonstrated better plant protection when the inoculated bacteria with improved rhizosphere-competence were used (Bonaldi et al., 2015). Colonization studies of *P. aeruginosa* BRp3 under field conditions using viable count showed that the strain had the potential to colonize on rice roots as well as on shoots up to 60 days (Figures 12, 13). Green colored colonies and pigmentation were the main characteristics of *P. aeruginosa* BRp3 that facilitated its detection from the roots and shoots of rice among indigenous soil populations under field conditions using viable count method. Colonization studied by IF and CLSM was in accordance with the results of viable counts of strain BRp3. It appeared that strain BRp3 colonized the rhizoplane and was also found as a shoot endophyte of rice plant. The number of most of the biological control agents decreases with time after their application in the environment, which affect the synthesis of inhibitory metabolites produced by these bacteria (Ji et al., 2008). Efficacy of the biocontrol agent depends on the proportion of root-colonized bacterial cells of the antagonistic bacteria. The influence of the introduced strain BRp3 on indigenous microbiota was estimated by comparing the total culturable bacterial population levels with those of different treatments under field conditions. Detection of higher number of culturable bacterial population indicated that BRp3 inoculation did not decrease the native bacterial population, which may be involved to a certain extent for promoting the plant growth (Susana et al., 2007).

To study plant-interacting bacteria, such as phytopathogens, symbionts, endophytes, biocontrol agents, and rhizospheric bacteria requires demonstration of re-infection and establishment of the inoculated strain in or on field-grown plants (Berg et al., 2014). Efficient root colonization by fluorescent *Pseudomonas* spp. has been reported to play an important role in their biocontrol activity against various plant pathogens (Bonaldi et al., 2015).

The significance of this study is that functionally characterized antagonistic *P. aeruginosa* BRp3 may be used for biocontrol of BLB along with enhanced rice growth. Even though, *Pseudomonas* spp. are indigenous and present in various rhizomicrobiomes but some of these can grow above 37°C and may become opportunistic pathogens, hence suitable biosafety regulations are needed to practically implement this technology for field application (Vilchez et al., 2016). *P. aeruginosa* BRp3 will be subjected to acute toxicity tests on mice to study the biosafety of this novel bacterial biocontrol agent. Absence of hemolytic activity on blood agar plates (Unpublished data) indicated that rhizospheric *P. aeruginosa* BRp3 may be unlikely to be a human pathogen (Radhapriya et al., 2015) but whole genome of this bacterium will be sequenced and compared with non-pathogenic *P. aeruginosa* strains like ATCC 15442 (Wang et al., 2014) to better understand the pathogenicity for its safe application.

## Conclusions

The study presents the detailed physiological characterization and effect of *P. aeruginosa* BRp3 inoculation on rice variety Super Basmati in the presence as well as in the absence of BLB pathogen. This bacterium produced a series of already characterized and novel analogs of HAQs, siderophores and rhamnolipids. The discovery of polyhydroxy HAQs may provide the insight about the biosynthesis pathway of these interesting compounds. The resistance was also induced in rice plants by BRp3 as the activity of defense related enzymes increased after pathogen inoculation. Collectively, the induction of defense related enzymes, HAQs along with siderophores in the presence of rhamnolipids and HCN, were responsible for the profound antibacterial activity of BRp3 against Xoo pathogen. The increased biomass production by strain BRp3 may be attributed to the production of IAA and siderophores. The colonization of the inoculated BRp3 and its re-isolation from rhizosphere indicated its better survival and rhizosphere-competence. On the basis of overall results achieved during this study, bacterial strain BRp3 may be an effective bio-inoculant for Super Basmati rice after ensuring its biosafety aspects. This is perhaps the first systematic effort to use functionally well-characterized beneficial *Pseudomonas* sp. capable of diverse secondary metabolite production for biocontrol of BLB pathogen in the country.

## Author contributions

SY was involved in conducting whole research work, data analysis, and write up. FH gave the basic idea to use beneficial bacteria as biocontrol agent and supervised the study. MM helped in 16S rRNA gene sequencing and edited the manuscript. MR helped in lab/field experiments and data analysis. HA provided reference strains of pathogen and involved in conducting experiments at NIAB. MZ helped in LC-MS analysis. MI did LC-MS analysis and edited the manuscript.

### Conflict of interest statement

The authors declare that the research was conducted in the absence of any commercial or financial relationships that could be construed as a potential conflict of interest.
