# Supplementary figures and images for: Biocontrol of Bacterial Leaf Blight of Rice and Profiling of Secondary Metabolites Produced by Rhizospheric Pseudomonas aeruginosa BRp3
Source: Front Microbiol. 2017 Sep 26;8:1895. doi: 10.3389/fmicb.2017.01895 (PMC5622989; doi:10.3389/fmicb.2017.01895)

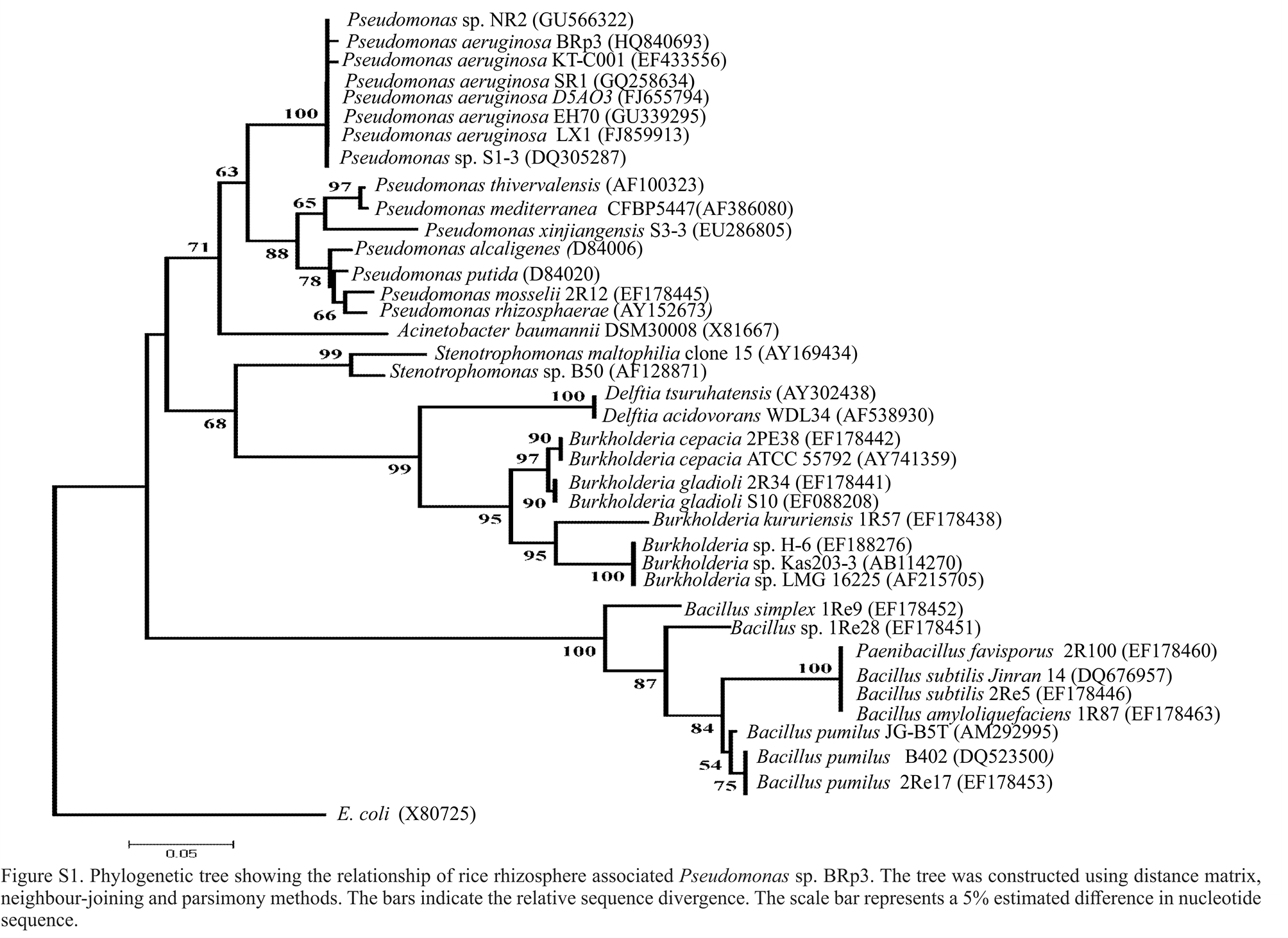

Supplement: Supplementary file 1 [file Image1.TIF]

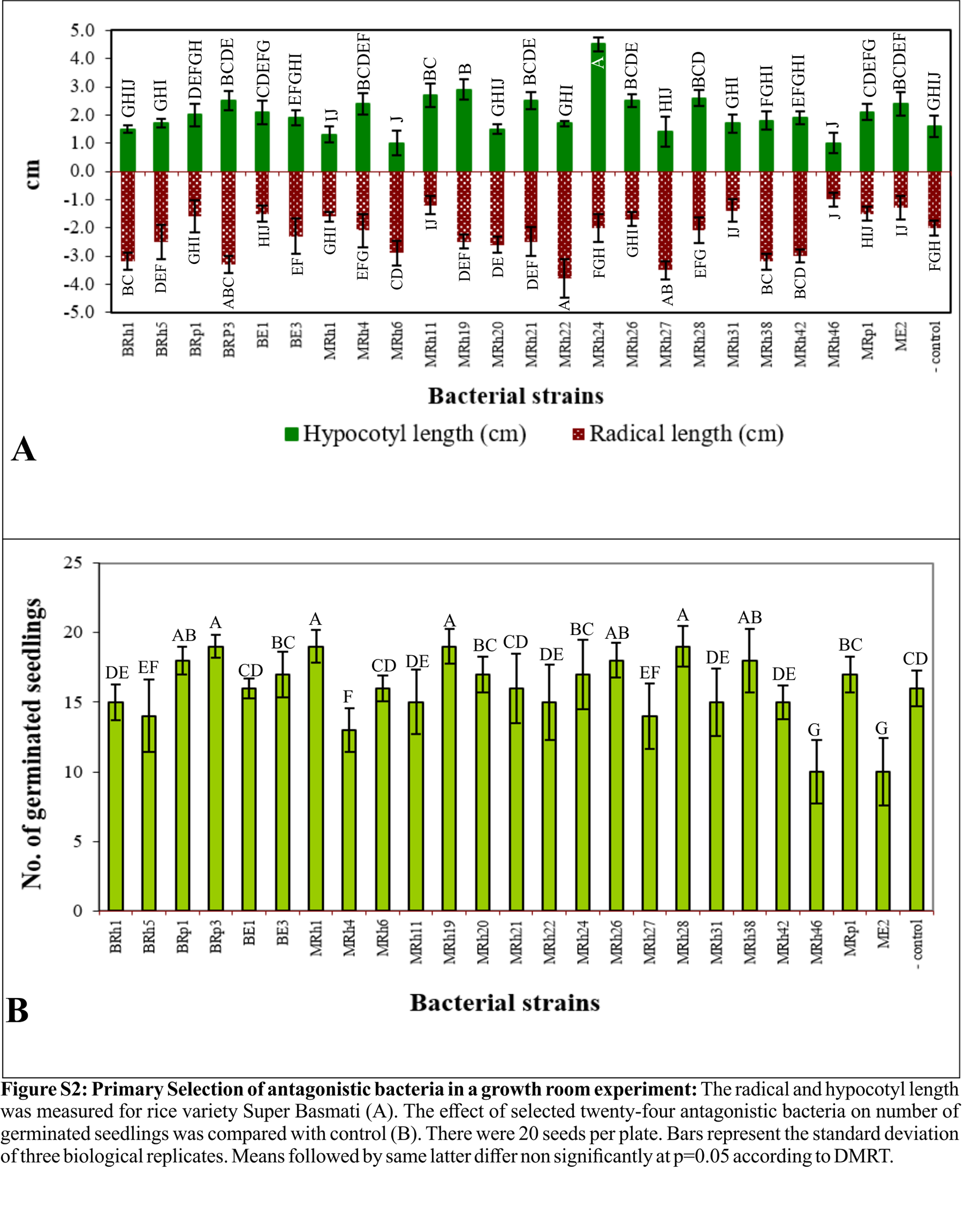

Supplement: Supplementary file 2 [file Image2.TIF]

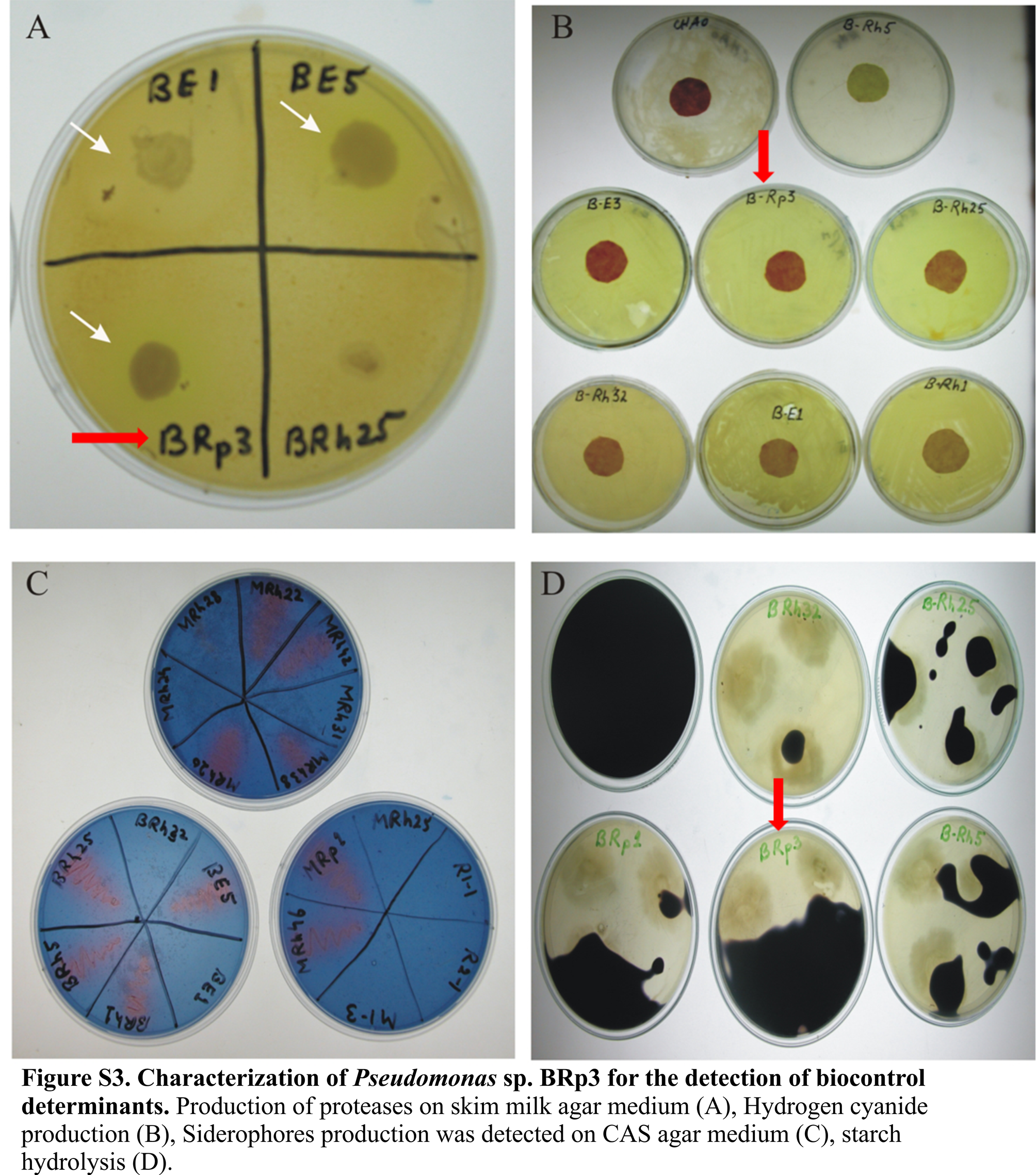

Supplement: Supplementary file 3 [file Image3.TIF]

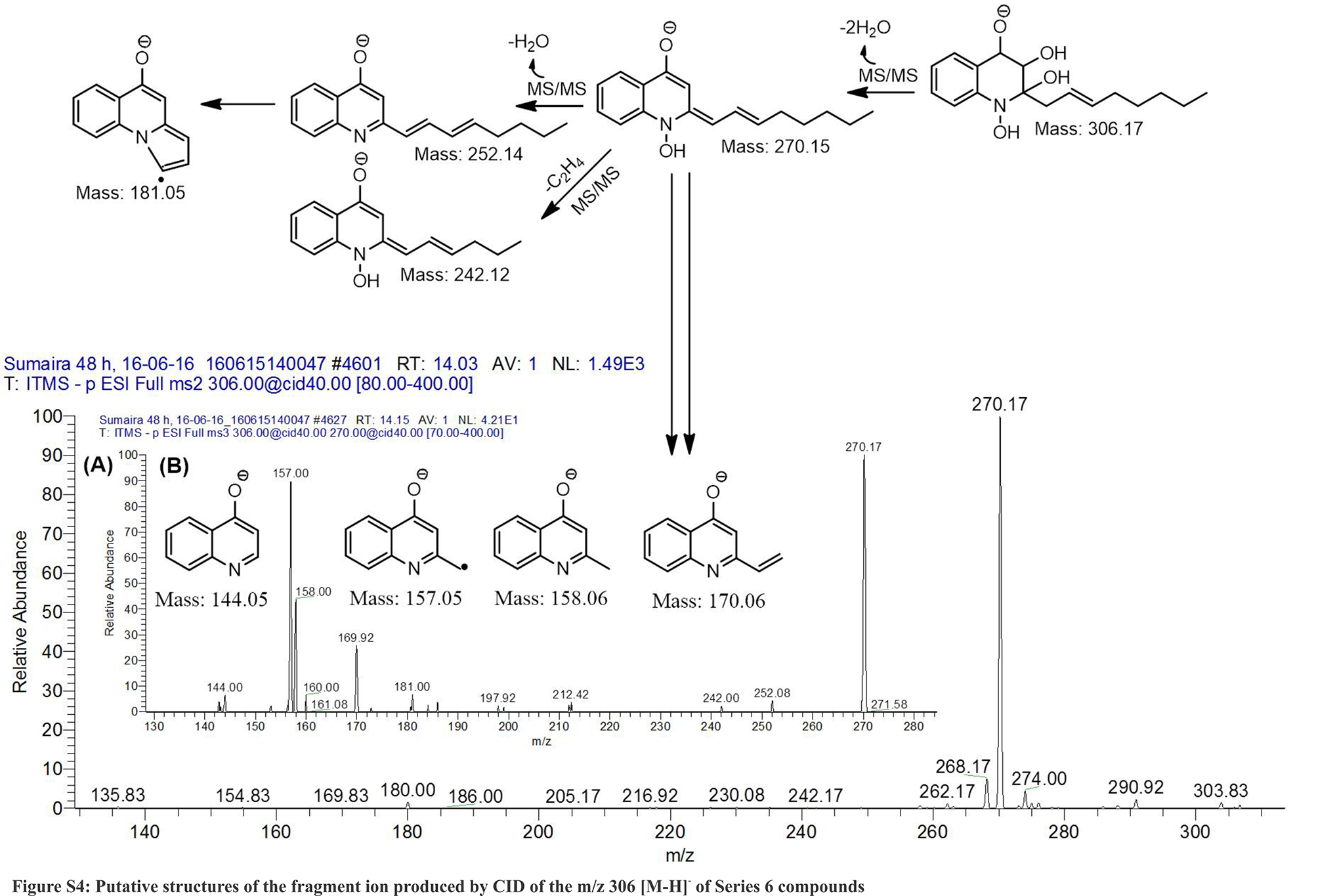

Supplement: Supplementary file 4 [file Image4.TIF]

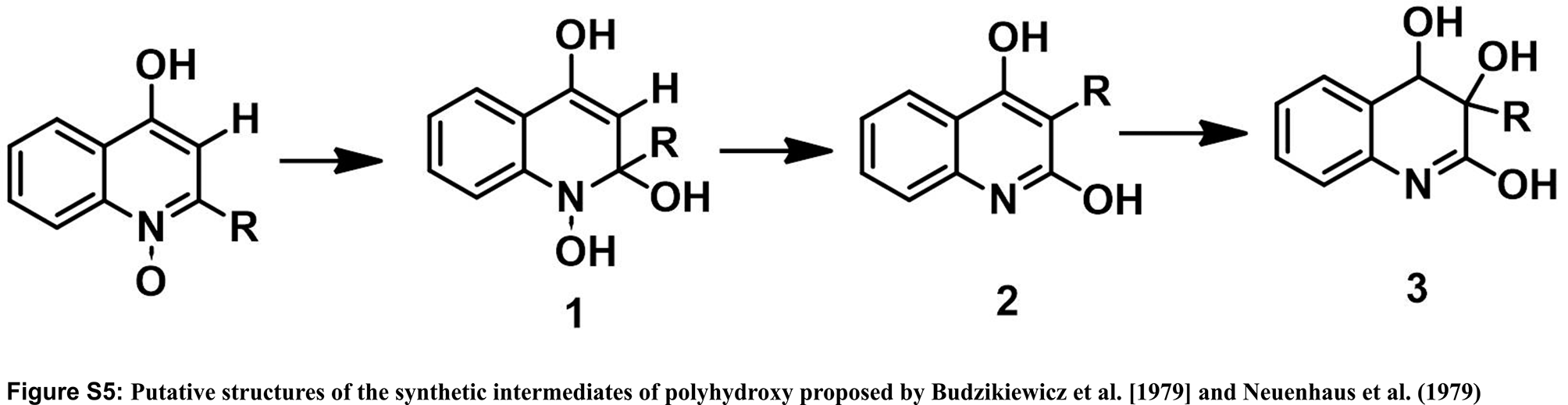

Supplement: Supplementary file 5 [file Image5.TIF]

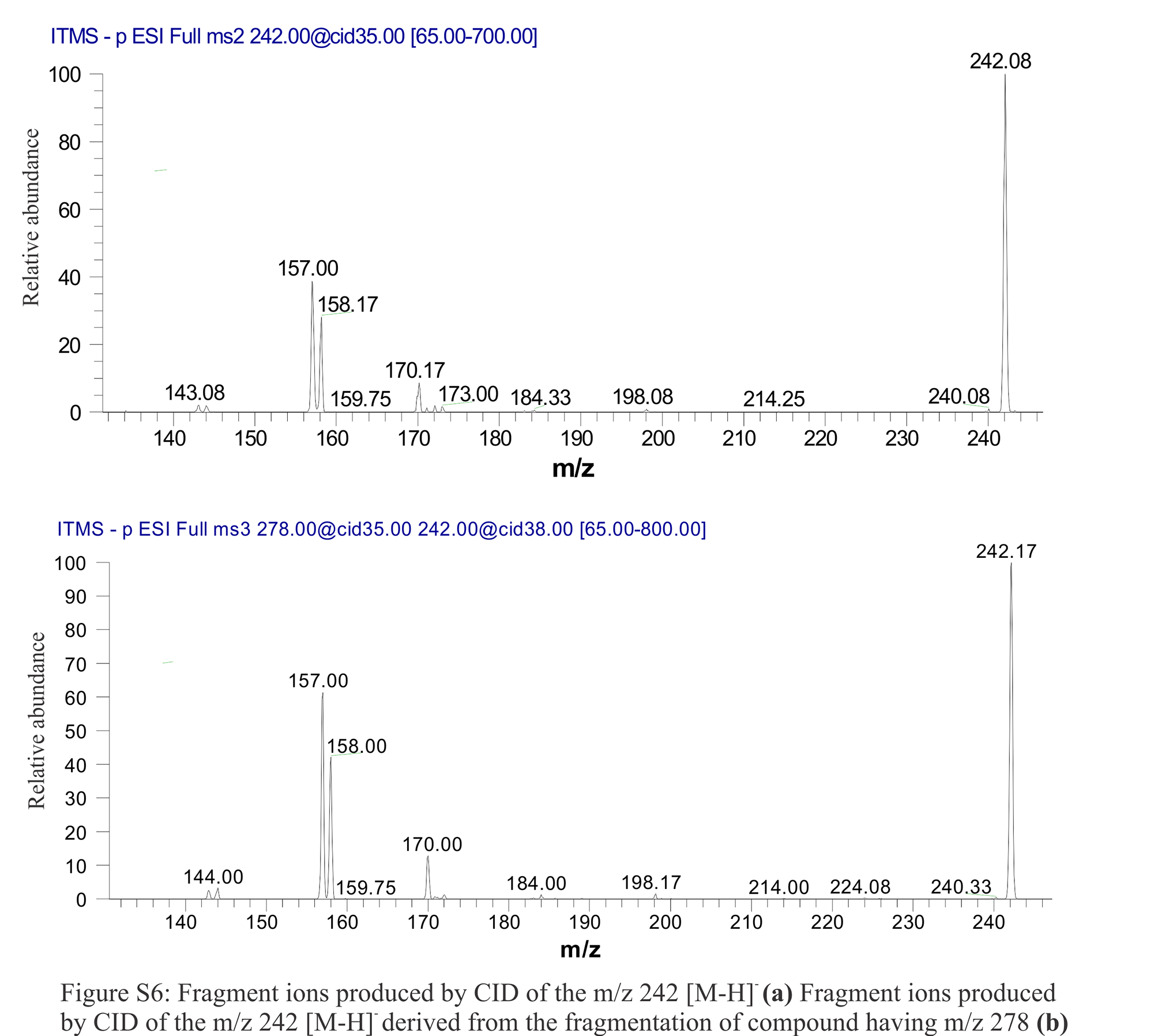

Supplement: Supplementary file 6 [file Image6.TIF]

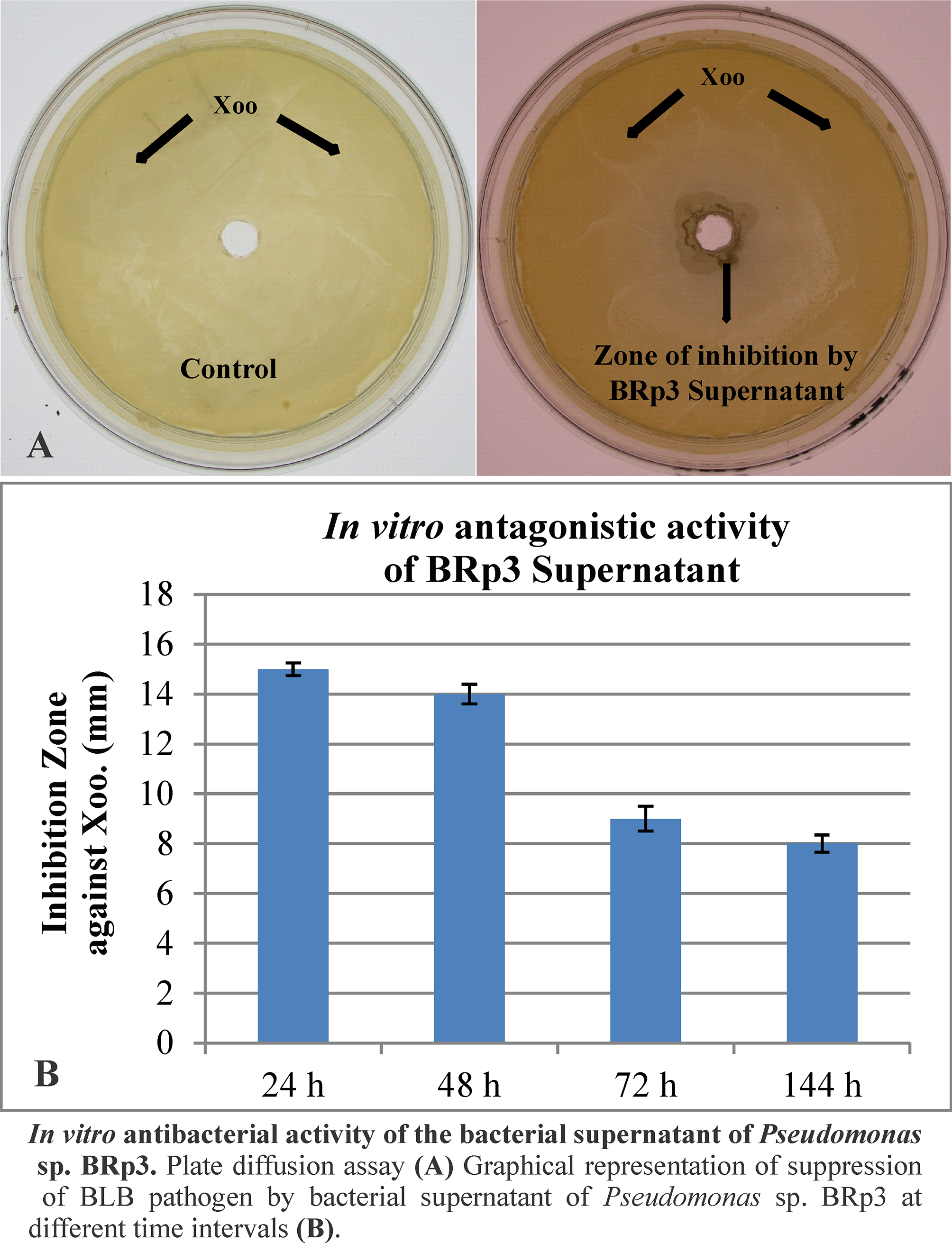

Supplement: Supplementary file 7 [file Image7.TIF]

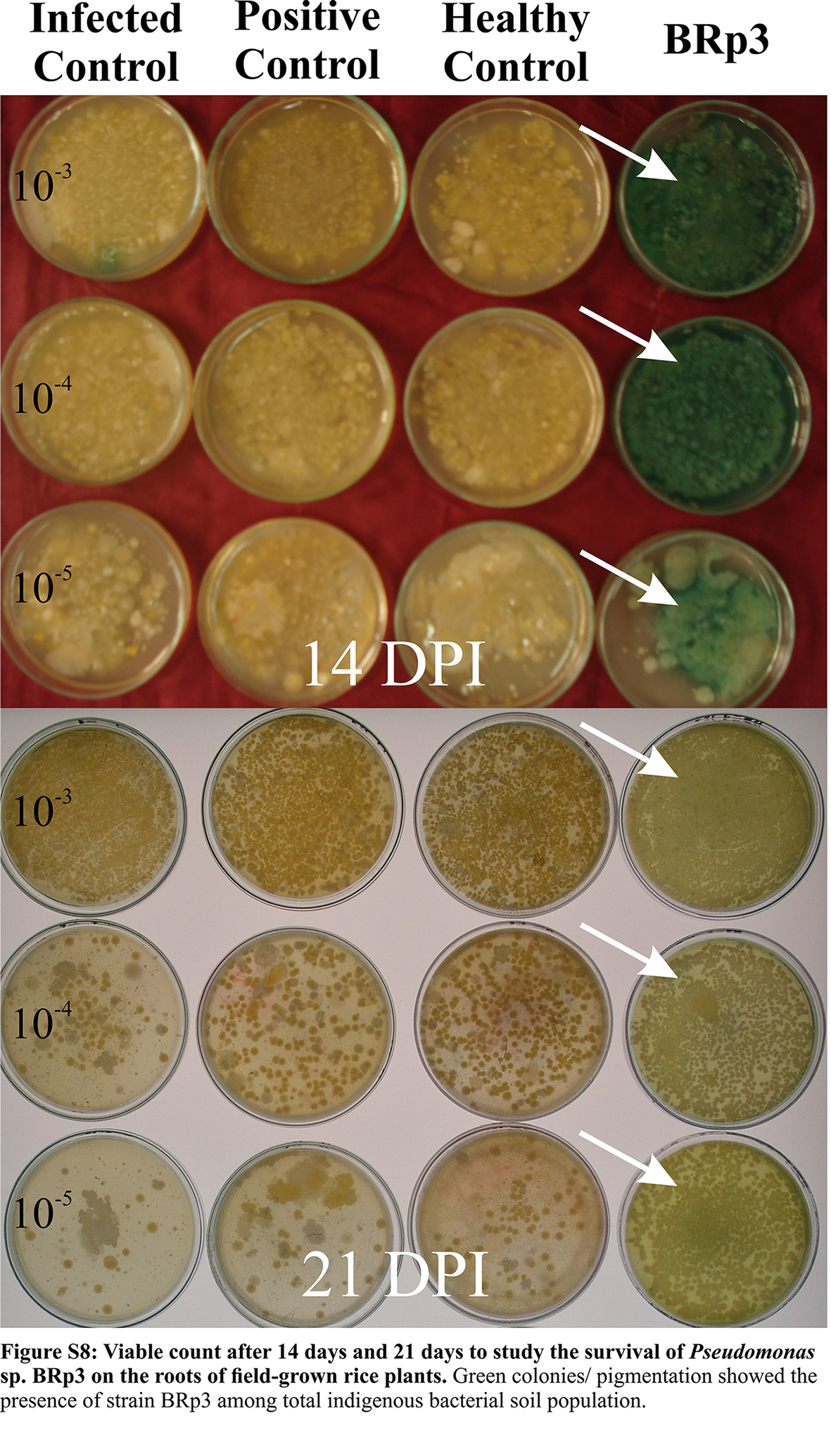

Supplement: Supplementary file 8 [file Image8.TIF]

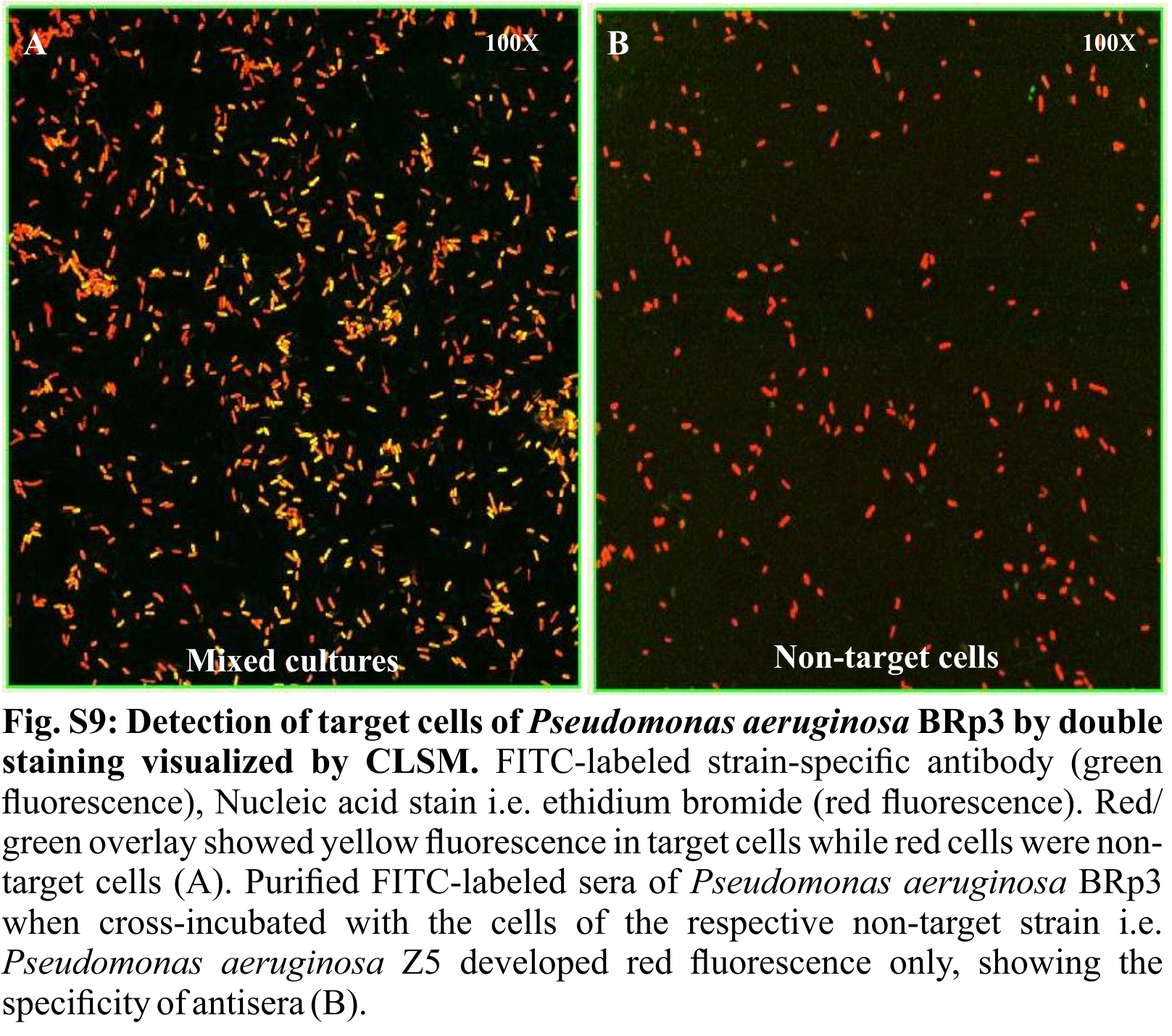

Supplement: Supplementary file 9 [file Image9.TIF]
