# Supplementary material for: Biocontrol of Bacterial Leaf Blight of Rice and Profiling of Secondary Metabolites Produced by Rhizospheric Pseudomonas aeruginosa BRp3
Source: Front Microbiol. 2017 Sep 26;8:1895. doi: 10.3389/fmicb.2017.01895 (PMC5622989; doi:10.3389/fmicb.2017.01895)
Supplement: Supplementary file 10 [file Table1.DOC]

**Table: Isolation and antagonism of bacterial strains/ isolates against bacterial/ fungal pathogens**

| **S.**  **No.** | **Strains/ isolates** | **Host** | **Medium used for isolation** | **Location**  **of isolation** | **Diameter of inhibition of**  ***Xanthomonas oryzae* pv. *oryzae* (Xoo) strains**  **Diffusion plate assay**  **(mm)** | | | | | | **% Inhibition against**  ***Fusarium* spp.**  **Dual culture assay** | | |
| --- | --- | --- | --- | --- | --- | --- | --- | --- | --- | --- | --- | --- | --- |
|  |  |  |  |  | **Xoo1** | **Xoo2** | **Xoo4** | **Xoo5** | **Xoo6** | **Xoo7** | ***F.***  ***oxysporum*** | ***F. monoliformae*** | ***F.***  ***solani*** |
|  | BRh1 | Rice | Nutrient agar | Lahore | *-* | *-* | 4±0.3 | *-* | 11±0.8 | 5±0.2 | 18.5±0.4 | 40.9±1.3 | 30±1.9 |
|  | BRh5 |  |  |  | - | - | - | 15± 0.4 | 14±0.6 | 12±1.0 | 14.8±0.3 | 45.5±2.5 | 30±2.4 |
|  | BRh6 |  |  |  | - | - | 6±0.5 | 5±0.1 | - | 6±0.2 | - | - | 54±4.3 |
|  | BRh18 |  |  | Sheikhupura | - | - | 5.4±0.6 | 8±0.3 | *-* | 7±0.1 | - | 15±1.6 | 14.3±0.7 |
|  | BRh19 |  |  |  | - | - | 5.2±0.4 | - | 5±0.2 | 9±0.2 | - | 16.7±1.3 | - |
|  | BRh21 |  |  |  | - | - | 4.7±0.3 | 3±0.2 | - | 10±0.3 | - | 35.9±2.3 | - |
|  | BRh25 |  |  |  | - | - | 3.9±0.2 | 9±0.2 | - | - | 56.7±3.5 | 76.7±7.6 | 46.2±4.6 |
|  | BRh27 |  |  | Faisalabad | - | - | 2.8±0.2 | - | - | 4±0.1 | - | 25.7±1.3 | - |
|  | BRh32 |  |  |  | - | - | 3.4±0.1 | - | - | 8±0.4 | 42.9±4.3 | 42.9±2.8 | 58.3±2.6 |
|  | BRh33 |  |  |  | - | - | 3.9±0.2 | *-* | *-* | 6±0.2 | - | - | 24.2±1.3 |
|  | BRp1 |  |  | Jhang | - | - | 4.2±0.3 |  | 5±0.2 | 9±0.4 | - | 20±1.9 | 25±2.2 |
|  | **BRp3** |  |  |  | 20±1.2 | 24± 1.5 | 10±1.0 | 10±1.0 | 20±1.4 | 12±1.4 | - | 43.3±1.3 | 75±4.3 |
|  | BRp4 |  |  |  | - | - | 5.3±0.2 | *-* | 2±0.1 | 7±0.2 | - | - | 24±1.5 |
|  | BRp5 |  |  | Gujranwala | - | - | 4.6±0.3 |  | 9±0.3 | 3±0.2 | 21±2.0 | - | 16±1.2 |
|  | BE1 |  |  |  | - | - | 4.7±0.2 | 5±0.2 | - | 8±0.2 | 76±5.3 | 50±2.7 | - |
|  | BE3 |  |  |  | - | - | 5.4±0.3 | 3±0.2 | - | 5±0.1 | 51.6±2.3 | 50±3.4 | 46.7±5.3 |
|  | BE5 |  |  |  | - | - | 12±1.0 | 18±1.5 | 20±1.0 | 12±1.2 | - | - | 15.2±1.1 |
|  | MRh1 | Cotton | *Pseudomonas* specific medium | Multan | - | - | 4±0.2 | - | - | 5±0.1 | 44.4±3.3 | - | - |
|  | MRh4 |  |  |  | - | - | 2.6±0.1 | - | 5±0.2 | 6±0.1 | 40±2.1 | - | - |
|  | MRh6 |  |  |  | - | - | 2.9±0.2 | - | - | 5±0.2 | 40±3.6 | 37.1±4 | - |
|  | MRh7 |  |  |  | - | - | 5±0.1 | - | - | 7±0.3 | - | 38.3±3.5 | - |
|  | MRh11 |  |  |  | - | - | 5.6±0.2 | - | - | 10±0.2 | 44.4±3.1 | 44.4±1.5 | - |
|  | MRh17 |  |  |  | - | - | 4.6±0.3 | - | - | 8±0.4 | - | 44.4±2.1 | - |
|  | MRh19 |  |  |  | 20±0.2 | 16± 1.2 | 15±1.5 | 11±1.2 | 10± 1.2 | 6±1.0 | - | - | - |
|  | MRh20 | Sugarcane | *Pseudomonas* specific medium | Faisalabad | 4±1.0 | - | 7±1.0 | - | 12±1.0 | 17±1.2 | 39.4±4.3 | 38.3±4.3 | - |
|  | MRh21 |  |  |  | - | - | 5±0.2 | - | 5±0.1 | 8±0.2 | 36.6±1.6 | - | - |
|  | MRh22 |  |  |  | 16±1.5 | - | 15±1.2 | 5±1.0 | 8±1.0 | 8±0.5 | 22.2±1.3 | - | - |
|  | MRh23 |  |  |  | - | - | 4±0.2 | - | 3±0.1 | 7±0.2 | 33.3±2.7 | - | - |
|  | MRh24 |  |  |  | - | - | 2.6±0.1 | - | 6±0.4 | 4±0.1 | 38.3±3.4 | - | - |
|  | MRh25 |  |  |  | - | - | 3.2±0.2 | - | - | 5±0.3 | 38.7±2.9 | - | - |
|  | MRh26 |  |  |  | - | - | 3.5±0.1 | - | 4±0.1 | 9±0.2 | - | 35.5±2.3 | - |
|  | MRh27 |  |  |  | - | - | 3.6±0.2 | - | 5±0.2 | 10±0.6 | 22.2±1.8 | - | - |
|  | MRh28 |  |  |  | - | - | 4.3±0.2 | 6±0.2 | - | 5±0.2 | 28.3±1.2 | 37.8±3.5 | - |
|  | MRh29 |  |  |  | - | - | 4.2±0.3 | - | 4±0.2 | - | - | 35.5±2.8 | - |
|  | MRh30 |  |  |  | - | - | 4.5±0.3 | - | 3±0.1 | - | - | 38.3±2.6 | - |
|  | MRh31 |  |  |  | - | - | 2.8±0.2 | 3±0.2 | - | 9±0.2 | 38.3±1.5 | 36±3.3 | - |
|  | MRh32 |  |  |  | - | - | 3.2±0.1 | - | 6±0.2 | 7±0.2 | - | 48.8±3.6 | - |
|  | MRh33 |  |  |  | - | - | 3.5±0.2 | 5±0.1 | - | 6±0.2 | 33.3±2.6 | - | - |
|  | MRh34 |  |  |  | - | - | 3.9±0.3 | - | - | 8±0.5 | - | - | - |
|  | MRh36 |  |  |  | - | - | 6±0.4 | - | 5±0.2 | - | - | - | 41.1±3.2 |
|  | MRh37 |  |  |  | - | - | 4.3±0.2 | - | 4±0.1 | 9±0.4 | - | - | - |
|  | MRh38 |  |  |  | 15±1.2 | 18±1.4 | 5±1.0 | 25±1.2 | 30±1.5 | 10±1.5 | - | 37.7±2.1 | 38±2.4 |
|  | MRh42 |  |  |  | - | 9±1.2 | 5±1.0 | 9±1.4 | 14±1.0 | 9±1.3 | 66.6±4.4 | - | 42.2±3.3 |
|  | MRh44 |  |  |  | - | - | 3±0.1 | - | 4±0.1 | 8±0.2 | - | - | 46.6±4.3 |
|  | MRh45 |  |  |  | - | - | 3.4±0.2 | 4±0.2 | - | 6±0.3 | - | - | - |
|  | MRh46 |  |  |  | - | - | 3.6±0.2 | - | 3±0.1 | 10±0.2 | 33.3±2 | - | - |
|  | MRp1 | Cotton | *Pseudomonas* specific medium | Multan | 13±1.5 | - | 6±1.4 | 4±1.2 | 15±1.5 | 10±1.4 | 35.5±2.5 | - | - |
|  | MRp2 |  |  |  | - | - | 4.1±0.2 | - | - | 7±0.2 | 39.6±2.4 | - | 55.5±3.5 |
|  | MRp4 |  |  |  | - | - | 3.4±0.2 | - | 6±0.1 | - | - | 36±2.3 | - |
|  | MRp7 |  |  |  | - | - | 3.7±0.2 | - | 4±0.2 | 6±0.2 | - | 37.1±3.9 | 45.5±3.6 |
|  | MRp8 |  |  |  | - | - | 3.6±0.2 | 3±0.2 | - | 8±0.4 | - | - | - |
|  | ME1 |  |  |  | - | - | 4.3±0.2 | - | - | 3±0.2 | - | 66.6±4 | - |
|  | ME2 |  |  |  | - | - | 4.5±0.1 | - | 4±0.1 | 9±0.3 | - | 34.4±2.1 | - |
|  | ME4 |  |  |  | - | - | 4.2±0.2 | - | 2±0.2 | 4±0.2 | - | 38.3±2.6 | - |
|  | Isolate  E 1 | BB resistant *Super Basmati*  Line 5 |  | NIAB, Faisalabad  (Isolated in the present study from BB resistant varieties of rice) | - | - | 2±0.2 | - | - | - | ND | ND | ND |
|  | Isolate E2 |  |  |  | - | - | 3.5±0.1 | - | - | - |  |  |  |
|  | Isolate  Rh1 | BB resistant *Super Basmati*  Line 4 |  |  | - | - | 4±0.2 | - | - | - |  |  |  |
|  | Isolate  E4 |  |  |  | - | - | 4.2±0.2 | - | - | - |  |  |  |
|  | Isolate  E5 |  |  |  | - | - | 3.7±0.3 | - | - | - |  |  |  |
|  | Isolate  E6 |  |  |  | - | - | 4.5±0.2 | - | - | - |  |  |  |
|  | Isolate  Rh7 | BB resistant *Super Basmati*  Line 22 |  |  | - | - | 3.2±0.2 | - | - | - |  |  |  |
|  | Isolate  Rh8 |  |  |  | - | - | 2±0.2 | - | - | - |  |  |  |
|  | Isolate  Rh9 |  |  |  | - | - | 2.9±0.1 | - | - | - |  |  |  |
|  | Isolate  Rh10 | BB resistant *Super Basmati*  Line 11 |  |  | - | - | 3.5±0.2 | - | - | - |  |  |  |
|  | Isolate  Rh11 |  |  |  | - | - | 4.8±0.3 | - | - | - |  |  |  |
|  | Isolate  Rh12 |  |  |  | - | - | 5±0.4 | - | - | - |  |  |  |
|  | R1-1 | (obtained from  NBRC  Culture Collection) |  | Swat | - | - | 12±0.2 | 15±0.8 | - | - | ND | ND | ND |
|  | R2-1 |  |  |  | - | - | 1.9±0.1 | - | 4±0.2 |  | ND | ND | ND |
|  | R5-2 |  |  |  | - | - | 2±0.1 | - | 5±0.2 | - | ND | ND | ND |
|  | R6-2 |  |  |  | - | - | 1.6±0.1 | - | 3.5±0.2 | - | ND | ND | ND |
|  | Mi |  |  | Faisalabad | - | - | 6±1.0 | 12+1.5 | 13+1.0 | 9+1.3 | ND | ND | ND |
|  | M1-3 |  |  | Bangladesh | - | - | 1.6±0.1 | - | 20±0.2 | - | - | - | - |
|  | M1-1 |  |  |  | - | - | 1.8±0.2 | - | 13±0.2 | - | - | - | - |
|  | WN1 |  |  | Pakistan | - | - | 1.8±0.2 | - | - | 10±0.3 | ND | ND | ND |
|  | Wb1 |  |  | Pakistan | - | - | 1.7±0.2 | - | - | 9±0.3 | ND | ND | ND |
|  | 8N-4 |  |  | Mongolia | - | - | 1.5±0.2 | - | 15±0.2 | 2±0.1 | 3±0.2 | - | - |
|  | Ms-3Y |  |  |  | - | - | 2±0.2 | 7±0.3 | 3±0.2 | 2±0.1 | ND | ND | ND |
|  | 4.2.1.A |  |  | Indonesia | - | - | 4±0.2 | 6±0.1 | 3±0.2 | - | ND | ND | ND |
|  | 5.1.A |  |  |  | - | - | - | 2±0.2 | 4±0.1 | - | ND | ND | ND |

Bacterial strains were isolated from rice and other host plants in this study and were also obtained from NIBGE Biotech Resource Centre (NBRC), Faisalabad, Pakistan

Name of bacterial strains with **R, Rh, Rp** and **E** indicated their origin of isolation from Root, Rhizosphere, Rhizoplane and Endosphere of their respective hosts, respectively.

**Dual culture assay** was performed *in vitro* on PDA plates against different pathogens

% **Percent Suppression** of Fungi calculated as = [1 – (Fungal growth / Control growth)] x 100

All values are an average of three biological replicates, ± standard deviation, ND (not determined)

Faisalabad, Gujranwala, Jhang, Multan, Sheikhupura and Swat are different cities of Pakistan. NIAB: Nuclear Institute for agriculture and Biology, Faisalabad-Pakistan.
