# Supplementary material for: Biocontrol of Bacterial Leaf Blight of Rice and Profiling of Secondary Metabolites Produced by Rhizospheric Pseudomonas aeruginosa BRp3
Source: Front Microbiol. 2017 Sep 26;8:1895. doi: 10.3389/fmicb.2017.01895 (PMC5622989; doi:10.3389/fmicb.2017.01895)
Supplement: Supplementary file 11 [file Table2.DOC]

**Table: Physiological characterization of rhizosphere associated antagonistic bacteria**

| **Sr.**  **No.** | **Isolates**  **code** | **Gram reaction** | **Catalase** | **Cytochrome oxidase** | **Growth promoting properties** | | **Biocontrol determinants** | | | |
| --- | --- | --- | --- | --- | --- | --- | --- | --- | --- | --- |
|  |  |  |  |  | **^1^IAA**  **(μg/mL)** | **^2^ P**  **(μg/mL)** | **^3^ Siderophores** | **^4^ Proteases** | **^5^ HCN** | **^6^ Starch**  **hydrolysis** |
| 1 | BRh1 | - | - | + | + | + | - | - | + | - |
| 2 | **BRh5** | - | + | + | 1.2±0.1 | 65±3.4 | ++ | - | - | +++ |
| 3 | BRh6 |  | + | + | + | - | - | - | - | + |
| 4 | BRh18 | - | + | + | - | ++ | - | - | - | + |
| 5 | BRh19 | - | - | + | - | ++ | - | - | - | - |
| 6 | BRh21 | - | + | - | - | ++ | - | - | ++ | + |
| 7 | BRh25 | - | + | + | - | - | - | - | ++ | +++ |
| 8 | BRh27 | - | + | + | - | - | - | - | - | ++ |
| 9 | BRh32 | - | + | + | - | - | - | - | ++ | +++ |
| 10 | BRh33 | - | - | + | - | - | - | - | - | - |
| 11 | BRp1 | + | + | + | + | + | - | - | - | ++ |
| 12 | **BRp3** | - | + | + | 30±2 | 97±4.1 | +++ | +++ | +++ | ++ |
| 13 | BRp4 | - | - | - | + | + | - | - | - | - |
| 14 | BRp5 | - | + | + | + | + | - | - | - | - |
| 15 | BE1 | - | - | - | + | - | - | - | + | - |
| 16 | BE3 | + | - | + | + | - | - | - | +++ | - |
| 17 | **BE5** | - | + | + | - | 72±5.2 | ++ | ++ | - | +++ |
| 18 | MRh1 | - | + | + | + | + | - | - | - | - |
| 19 | MRh4 | - | + | + | + | + | - | - | ++ | - |
| **Sr.**  **No.** | **Isolates**  **code** | **Gram reaction** | **Catalase** | **Cytochrome oxidase** | **Growth promoting properties** | | **Biocontrol determinants** | | | |
|  |  |  |  |  | **^1^IAA**  **(μg/mL)** | **^2^ P**  **(μg/mL)** | **^3^ Siderophores** | **^4^ Proteases** | **^5^ HCN** | **^6^ Starch**  **hydrolysis** |
| 20 | MRh6 |  | + | + | - | +++ | - | - | +++ | - |
| 21 | MRh7 | + | + | - | - | - | - | - | - | - |
| 22 | MRh11 | + | + | + | +++ | - | - | - | - | - |
| 23 | MRh17 | - | + | + | +++ | ++ | - | - | - | - |
| 24 | **MRh19** | - | + | + | - | - | ++ | - | ++ | - |
| 25 | **MRh20** | - | + | + | - | - | + | ++ | +++ | +++ |
| 26 | MRh21 | - | + | + | - | - | - | - | - | - |
| 27 | **MRh22** | - | + | + | 14±0.3 | - | + | ++ | - | + |
| 28 | MRh23 | + | + | - | +++ | - | - | - | - | - |
| 29 | MRh24 | - | + | + | - | - | - | - | - | - |
| 30 | MRh25 | - | + | - | +++ | - | - | - | ++ | - |
| 31 | MRh26 | - | + | + | - | - | - | - | - | - |
| 32 | MRh27 | - | - | + | - | - | - | - | - | - |
| 33 | MRh28 | + | - | + | - | - | - | - | - | - |
| 34 | MRh29 | - | - | + | - | - | - | - | - | - |
| 35 | MRh30 | - | - | + | +++ | - | - | - | +++ | - |
| 36 | MRh31 | - | + | + | - | +++ | - | - | +++ | - |
| 37 | MRh32 | - | ND | ND | - | - | - | - | - | - |
| 38 | MRh33 | - | + | + | +++ | - | - | - | ++ | - |
| 39 | MRh34 | - | ND | ND | - | - | - | - | ++ | - |

| **Sr.**  **No.** | **Isolates**  **code** | **Gram reaction** | **Catalase** | **Cytochrome oxidase** | **Growth promoting**  **properties** | | **Biocontrol determinants** | | | |
| --- | --- | --- | --- | --- | --- | --- | --- | --- | --- | --- |
|  |  |  |  |  | **^1^IAA**  **(μg/mL)** | **^2^ P**  **(μg/mL)** | **^3^ Siderophores** | **^4^ Proteases** | **^5^ HCN** | **^6^ Starch**  **hydrolysis** |
| 40 | MRh36 | - | ND | ND | +++ | - | - | - | - | - |
| 41 | MRh37 | - | ND | ND | - | + | - | - | ++ | - |
| 42 | **MRh38** | - | + | + | - | - | ++ | - | - | + |
| 43 | **MRh42** | - | + | + | 5.2±0.8 | - | +++ | + | - | +++ |
| 44 | MRh44 | - | + | + | - | - | - | - | - | - |
| 45 | MRh45 | - | + | + | - | - | - | - | - | - |
| 46 | MRh46 | - | - | + | - | - | - | - | - | - |
| 47 | **MRp1** | - | + | + | 4.6±0.4 | - | ++ | ++ | ++ | + |
| 48 | MRp2 | - | + | + | +++ | - | - | - | ++ | - |
| 49 | MRp4 | - | ND | ND | - | - | - | - | - | - |
| 50 | MRp7 | - | ND | ND | - | - | - | - | - | - |
| 51 | MRp8 | - | ND | ND | - | - | - | - | - | - |
| 52 | ME1 | - | + | - | - | ++ | - | - | - | - |
| 53 | ME2 | - | + | + | - | - | - | - | - | - |
| 54 | ME4 | - | ND | ND | - | - | - | - | - | - |
| 55 | **Mi** | - | + | + | - | 50±4.7 | + | +++ | - | +++ |

Bacterial strains 1-17 were isolated on NB medium while bacterial strains 18-54 were isolated on medium (King’s B/ S1 ) specific for *Pseudomonas* spp.

**^1^** **Indole acetic acid** detected qualitatively by spot test and quantified by HPLC. **^2^** **Phosphate solubilization**: + represents clear halo zone <5 mm on Pikovskaia agar; ++ represents halo zone = 5 mm on Pikovskaia agar; +++ represents halo zone >5 mm on Pikovskaia agar; - represents no halo zone on Pikovskaia agar, P solubilization was quantified using spectrophotometer. **^3^** **Siderophore production** was detected on CAS agar. **^4^ Protease production** was carried out on skim milk agar medium. + represent < 5mm wide halo, ++ represent 5-10 mm halo zone, +++ represents > 10 mm halo zone. **^5, 6^** **Hydrogen cyanide (HCN) production** and **starch hydrolysis** was detected by plate assay, - represents no production, ++ represents hydrolysis of starch in more than half plate, +++ represents complete hydrolysis of starch in plate.

All the observations were recorded by repeating experiment thrice with three replicates each time, ± standard deviation
