# Supplementary material for: Biocontrol of Bacterial Leaf Blight of Rice and Profiling of Secondary Metabolites Produced by Rhizospheric Pseudomonas aeruginosa BRp3
Source: Front Microbiol. 2017 Sep 26;8:1895. doi: 10.3389/fmicb.2017.01895 (PMC5622989; doi:10.3389/fmicb.2017.01895)
Supplement: Supplementary file 12 [file Table3.DOCX]

**Table 3.3 Antibiotic resistance pattern of *Pseudomonas aeruginosa* strain BRp3**

| **S. No.** | **Antibiotic** | *** Conc.**  (µg/ mL) | **BRp3** |
| --- | --- | --- | --- |
| 1 | Amikacin | 30 | R |
| 2 | Aztrreonum | 30 | R |
| 3 | Ampicillin | 10 | R |
| 4 | Carbenicillin | 100 | R |
| 5 | Ceftriaxone | 30 | S |
| 6 | Cephradine | 30 | R |
| 7 | Chloramphenicol | 30 | R |
| 8 | Cefixime | 5 | S |
| 9 | Ciprofloxacin | 5 | S |
| 10 | Doxycycline | 30 | R |
| 11 | Erythromycin | 30 | R |
| 12 | Erytomicin | 15 | S |
| 13 | Gentamycin | 10 | R |
| 14 | Kanamycin | 30 | S |
| 15 | Neomycin | 30 | R |
| 16 | Nalidixic acid | 30 | R |
| 17 | Paratam | 75 | S |
| 18 | Ofloxacin | 5 | S |
| 19 | Rifampicin | 5 | S |
| 20 | Streptomycin | 10 | R |
| 21 | Trimethoprim | 25 | S |
| 22 | Tetracycline | 30 | S |

* Concentration of antibiotic used, R: Resistant, S: Susceptible
